# Distinct foliar fungal communities in *Pinus contorta* across native and introduced ranges: evidence for context dependency of pathogen release

**DOI:** 10.1038/s41598-025-91639-z

**Published:** 2025-03-01

**Authors:** Ruirui Zhao, Susan J. Nuske, Martín A. Nuñez, Alex Fajardo, Jaime Moyano, Anne C. S. McIntosh, Marie-Charlotte Nilsson, Michael J. Gundale

**Affiliations:** 1 Department of Forest Ecology and Management, Swedish University of Agricultural Sciences, Umeå, SE-90183 Sweden; 2 EcoFutures, Brisbane, 4006 Australia; 3 Grupo de Ecología de Invasiones, INIBIOMA-UNComa, CONICET, Bariloche, AR-8400 Argentina; 4 Department of Biology and Biochemistry, University of Houston, Houston, TX-77204 USA; 5 Dirección de Investigación, Vicerrectoría Académica, Universidad de Talca, Talca, 3460000 Chile; 6 Instituto de Ecología y Biodiversidad (IEB), Las Palmeras 3425, Ñuñoa, 7750000 Chile; 7 Science Department, Augustana Faculty, University of Alberta, Camrose, AB T4V 2R3 Canada

**Keywords:** Lodgepole pine, Foliar microbiota, Pathogens, Biogeographical variation, Tree invasion, Enemy release hypothesis, Forest ecology, Invasive species

## Abstract

Inter-continental study systems are crucial for testing ecological hypotheses, such as the widely cited Enemy Release Hypothesis (ERH), which seeks to explain the superior performance of plant species when they are introduced to new regions. *Pinus contorta* (lodgepole pine), native to North America, has been extensively introduced to Europe and the Southern Hemisphere, making it an ideal tree species for studying invasion hypotheses from a biogeographical perspective. We compared foliar fungal communities, especially pathogens, of *P. contorta* across two native–introduced region pairs (NIRPs): a northern NIRP (from Canada to Sweden) and a southern NIRP (from the USA to Patagonia), while also examining the differences between source plantations and invasion fronts within Patagonia. *P. contorta* underwent significant fungal community shifts and experienced pathogen release during its large-scale introduction from North America to Sweden and Patagonia. The fungal richness and relative abundance changes were more pronounced for the southern NIRP pair, where no closely related tree species to *P. contorta* are present in Patagonia. In Sweden, the presence of the phylogenetically related *P. sylvestris* and its associated local fungal community appears to play a role in influencing the foliar fungal communities associated with introduced *P. contorta*. In Patagonia, the incomplete co-invasion of fungal taxa from the USA emerges as a principal driver of the observed variability in fungal community composition and pathogen release following the introduction of *P. contorta*. In Patagonia, fungal community composition differences between source plantations and invasion fronts provided insufficient evidence that pathogen release occurs at this local scale. Integrating both biogeographical and phylogenetic perspectives, our study suggests that priority effects of local fungi appear to be a dominant community assembly process when introduction is done in a phylogenetically similar community; whereas, co-invasion of fungal communities is the dominant process in phylogenetically distant communities.

**Supplementary Information:**

The online version contains supplementary material available at 10.1038/s41598-025-91639-z.

## Introduction

Over the past century, fast-growing non-native tree species have been extensively introduced to new environments across the globe, where they often demonstrate superior performance, such as increased growth rates and larger individual sizes compared to those in their native habitats^1,2^. The foliar biological communities associated with introduced tree species play a pivotal role in establishing interactions with local ecosystems, contributing to pest and disease dynamics, facilitating resistance and adaptability, contributing to decomposition after senescence^3,4^, and ultimately influencing the successful establishment of these species within new ecological contexts^5,6^. For example, Otsing, et al.^3^ demonstrated that the community structure of all fungi, saprotrophs, and plant pathogens in foliage shapes leaf litter decomposition in mixed forest ecosystems. Moreover, interactions between pathogens and other fungal functional groups can also shape disease dynamics and, consequently, the successful introduction of tree species to new regions. For example, fungal saprotrophs associated with American beech (*Fagus grandifolia*) have been shown to influence the progression of beech bark disease^7^. Given the known importance of foliar fungal communities for tree performance, investigating how the tree introduction and subsequent potential invasion change the composition of foliar fungal communities can deepen our understanding of tree success following their introduction to new environments.

Numerous hypotheses have been proposed to explain the success of introduced plant species, with the Enemy Release Hypothesis (ERH) being particularly influential^8^. According to ERH, introduced plants can escape the specialist herbivores and pathogens in their native range^9,10^, and this reduced biotic stress allows resources that would typically be allocated to defense and recovery to be redirected toward biomass accumulation, resource acquisition, competition, and reproduction in the introduced range^11,12^. However, ecological factors, such as the environmental adaptability of introduced plant species and their phylogenetic relatedness to native species can significantly shape the relevance of ERH. For instance, during the introduction phase, species that share close phylogenetic relationships with native species may remain vulnerable to specialist pathogens due to similar host traits, whereas species that are phylogenetically distant from the local community may experience a greater release from natural enemies, as they are less likely to host specialist pathogens^13,14^. Moreover, even within the same region, pathogen transmission can exhibit spatial heterogeneity, with pathogens sometimes lagging behind their hosts, such as from source plantations to invasion fronts^15^. Given these complex influences, biogeographical studies comparing the communities of enemies in a species’ introduced versus native regions have emerged as one of the most compelling approaches to addressing invasion hypotheses such as ERH^16–18^. Further, on a local scale, a comprehensive investigation of exotic species’ fungal communities, especially pathogens, between source plantations and adjacent invasion fronts is also essential for evaluating the spatial and temporal scales over which ERH occurs^19^. *Pinus contorta* (lodgepole pine), native to western North America, stands out as a very successful introduced species in both Europe (e.g., Sweden, Scotland, Finland, and Ireland) and the Southern Hemisphere (e.g., Chile, Argentina, and New Zealand)^20–23^. In these introduced regions, *P. contorta* typically exhibits faster growth, earlier maturity, and greater reproductive output than in its native regions^17,24^. These traits facilitate its successful establishment and growth in the introduced ranges^25,26^, and in the Southern Hemisphere, they contribute to its success as an aggressive invader^27^. Given its extensively documented history of introduction, *P. contorta* represents an ideal “model” study system for investigating ecological hypotheses through a biogeographical perspective^28^. A key advantage of this large-scale study system is that the taxonomic composition of the communities into which *P. contorta* has been introduced varies substantially. In Sweden, *P. contorta* was introduced into environments dominated by native conifers, including *P. sylvestris*, a phylogenetically similar species^18,29^. In contrast, in the Southern Hemisphere, such as in Patagonia, no native *Pinus* species are present in the regions where *P. contorta* has been introduced^18,21^. Consequently, the *P. contorta* introduction study system represents an excellent opportunity for investigating the ERH across a diverse array of regions with varying levels of phylogenetic relatedness.

Since the introduction of *P. contorta* to Europe and the Southern Hemisphere in the 1970s, research has focused on the trees’ adaptability^30,31^, productivity^21,32^, invasiveness^25,26,33^, impact on local diversity^34–36^, and plant-soil feedback mechanisms^18,37^. However, no research has yet evaluated how introduction and invasions correspond with shifts in the foliar fungal communities, including pathogens. Given the diverse range of community contexts that *P. contorta* has been introduced to, investigating its foliar fungal communities has the potential to advance our understanding of key hypotheses that seek to explain exotic species’ success (e.g., ERH), and other microbial community assembly processes, and also to shed new light on the role these foliar microbial communities may play in explaining the higher productivity of *P. contorta* in its introduced compared to native environments^17,21^.

**Fig. 1:**
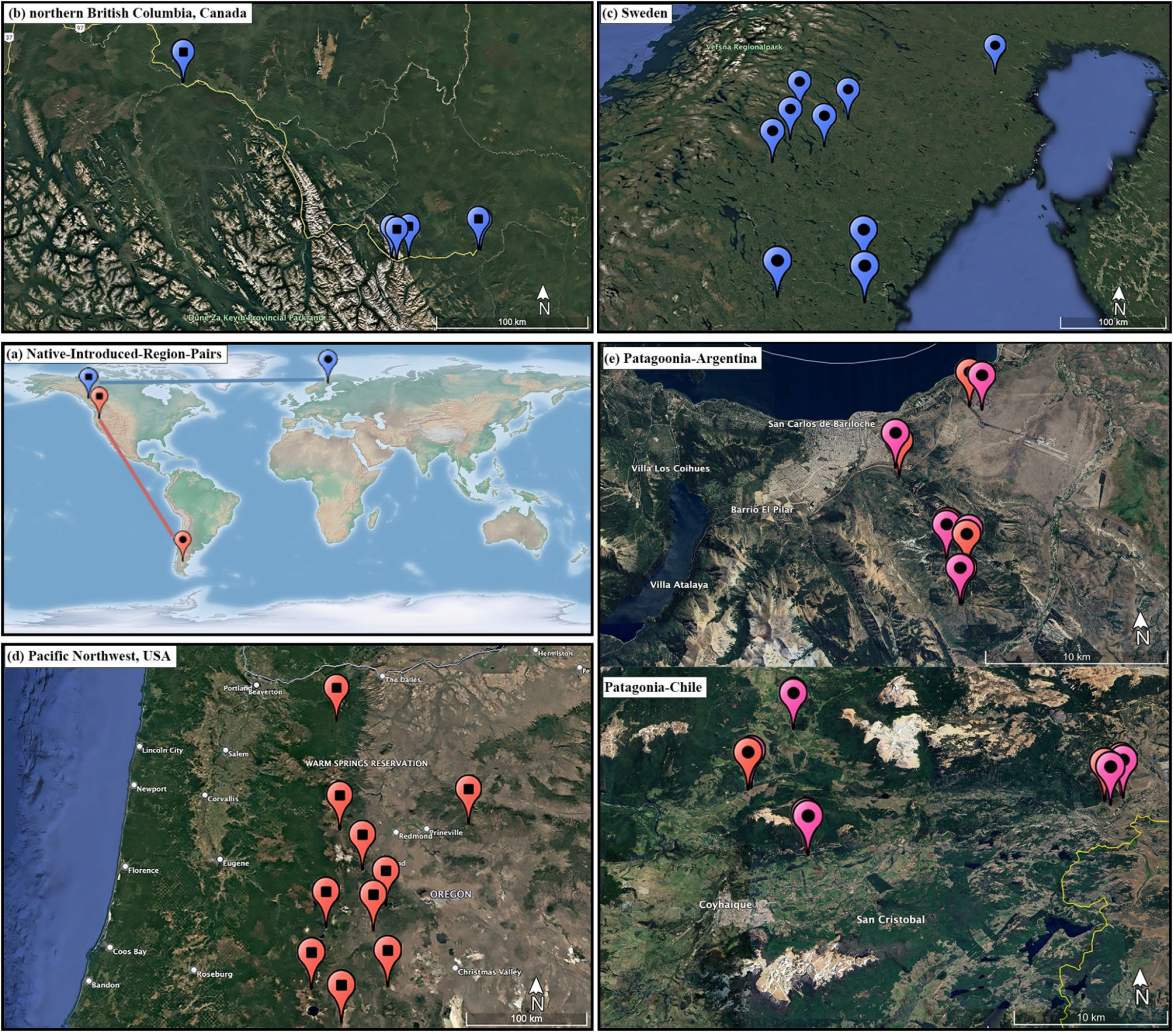
A map depicting two *Pinus contorta* native-introduced region pairs (NIRPs, panel **a**). The northern NIRP is represented in blue, indicating native range locations in northern British Columbia, Canada (**b**), and corresponding introduced populations in Sweden (**c**). The southern NIRP is depicted in red, indicating native range populations in the Pacific Northwest, USA (**d**), and corresponding introduced populations in Patagonia (**e**). In Patagonia, two paired stand types (i.e., plantations and invasion fronts) were sampled and represented in red and pink, respectively.

Our study aimed to investigate the foliar fungal community differences in the globally introduced *P. contorta* across its native (i.e., Canada and the USA) and introduced regions (i.e., Sweden and Patagonia), with a particular emphasis on assessing the occurrence of pathogen release during its introduction. Additionally, for the highly invasive Patagonia population, we further explored how fungal communities and associated pathogens shifted as *P. contorta* expanded from source plantations to invasion fronts over kilometer spatial scales. We employed *P. contorta* populations from two distinct native-introduced region pairs (NIRPs). In the northern NIRP, *P. contorta* was introduced from northern British Columbia, Canada, to Sweden, while in the southern NIRP, the species was introduced from the Pacific Northwest, USA, to Patagonia (Fig. 1)^18,38^. We tested the following hypotheses: After a large-scale introduction to new regions,

(H1a): *P. contorta* will exhibit shifts in composition and reduced richness of the whole foliar fungal community, as well as pathogens specifically (i.e., ERH), and

(H1b): This change will be more pronounced in the southern compared to northern NIRP, due to *P. contorta*’s greater phylogenetic difference from the dominant native community in Patagonia, which we anticipated would limit the pool of suitable local taxa.

(H2): In Patagonia, individuals at the invasion fronts will demonstrate a shifted foliar fungal community, reduced fungal richness, and experience pathogen release compared to the introduced plantations. We expected this because previous research has shown that invading *P. contorta* trees have a markedly lower root endophytic and ectomycorrhizal community diversity, suggesting that fungal co-invasion lags tree dispersal^18,39,40^.

## Materials and methods

### Study sites

For our study, we selected 40 sampling sites: 10 in Northern British Columbia, Canada; 10 in Sweden; 10 in the Pacific Northwest, USA; and 10 in the Patagonia region (i.e., Argentina and Chile). The distances between sampling plantation sites were 0.6–321.9 km in Canada, 1.7–57.7 km in Sweden, 0.6–321.7 km in the USA, and 0.8–179.4 km in Patagonia. Specifically, within Patagonia, plantation distances ranged from 0.8 to 5.7 km in Argentina and 1.9–39.9 km in Chile. Further, for the 10 sites in Patagonia, we identified adjacent invasions extending from plantations (referred to as “plantations” or “invasion fronts”), with the distances between invasion fronts and their paired adjacent plantations ranging from 0.1 to 0.9 km in Argentina and from 0.05 to 0.6 km in Chile (Fig. 1). In contrast, the 30 sites in North America and Sweden included only a single stand type: natural-origin stands in North America and introduced plantations in Sweden. The establishment of the study system began by identifying *P.* *contorta* plantations between 30 and 55 years of age in Sweden and the Southern Hemisphere^18^. The sites also represent native and introduced populations of two distinct subspecies, where *P. contorta* subsp. *latifolia* from Fort Nelson and Fort St. John British Columbia, Canada^38^ was introduced to Sweden and *P. contorta* subsp. *murrayana* from Central Oregon, USA was introduced to Patagonia. We refer to these as two distinct native-introduced region pairs (NIRPs, as mentioned above): a northern and a southern pair (Fig. 1). For introduced ranges, commercial plantations of *P. contorta* were extensively established in the 1970s. In Sweden, the 10 *P. contorta* subsp. *latifolia* stands were initiated as field trials in 1970 ^38^. The precise seed origin of Patagonia plantations is not known, but trait and anecdotal analysis have indicated the *P. contorta* subsp. *murrayana* population in central Oregon, USA as the origin^41^, where we distributed our native range sampling (Fig. 1).

### Needle sampling

During the 2019 growing season (July in the Northern Hemisphere and January in Patagonia), we randomly selected and sampled needles from eight trees in each stand, ensuring that the trees were evenly spaced. From each tree, we sampled sun-exposed branches within reach using long-handled pruning shears and subsequently collected needles, including old diseased, old healthy, young diseased, and young healthy needles (with “old” referring to 2-year-old needles and “young” to current-year growth). Needles were combined into one sample per tree, resulting in 397 needle samples in total. Upon collection, the needles were immediately placed in paper bags and frozen. Samples in Canada, the USA, and Patagonia were freeze-dried before they were shipped to the Forest Vegetation Ecology laboratory in Umeå, Sweden for further processing. In the laboratory, the needles were cleaned using deionized water and 0.5% Tween 20 to remove surface adherents before DNA extraction. For details about DNA extraction, PCR, and PacBio sequencing see Supplementary Methods.

### Bioinformatics analysis

After sequencing, data were processed using the nf-core collection of workflows [nf-core/ampliseq version 2.7.1^42,43^], which relies on reproducible software environments provided by the Bioconda^44^ and Biocontainers^45^ projects. Data quality was evaluated with FastQC (https://www.bioinformatics.babraham.ac.uk/projects/fastqc/) and summarized with MultiQC^46^. Primers and adapters were trimmed using Cutadapt^47^, and reads without matching primers or adapters were discarded. The adapter- and primer-free sequences were then processed on a per-sample basis with DADA2^48^, which corrected sequencing errors, removed chimeric sequences, and inferred true biological sequences, resulting in the generation of high-resolution amplicon sequence variants (ASVs). Finally, taxonomic annotation was performed and ASVs were assigned to corresponding taxa using the UNITE fungal database (Version 9.0)^49^.

After filtering, all downstream analyses, including plotting, were conducted using the statistical software R 4.4.0^50^. Firstly, each sample was rarified to 1000 sequence reads using the “rrarefy” function in the “vegan” package^51^. Then ITS ASVs were assigned functional guild annotations using FUNGuild^52^ through the “FUNGuildR” package, accepting all “probable” or higher-level annotations. We adopted the classifications of “plant pathogen”, “endophyte”, “epiphyte”, and “saprotroph”. Unidentified ASVs were consistently categorized as “unknown ecology”, and groups that fell outside the scope of our research focus (e.g., lichen, parasites, animal pathogens, etc.) were collectively classified as “others”. When an ASV was assigned to multiple functional groups, it was counted separately within each relevant group. For example, if an ASV was identified as “endophyte- leaf saprotroph- plant pathogen”, it was assigned to each of the corresponding categories: “endophyte”, “saprotroph”, and “plant pathogen”. The relative abundance of each functional group was defined as the total fractional abundance of all ASVs in that functional group, and richness was defined as the number of ASVs identified per sample per 1000 reads.

### Statistical analysis

For each of our hypotheses, forest stands served as the units of replication, and all univariate data were assessed for adherence to parametric assumptions (i.e., normality and homoscedasticity). All permutational multivariate analyses of variance (PERMANOVA) and tests for homogeneity of multivariate dispersion were performed using the “adonis2” and “betadisper” functions, respectively, from the “vegan” package. When significant interaction effects were detected (α = 0.05), post-hoc pairwise comparisons were made. For our first hypothesis, two-way PERMANOVA with “introduction status” (IS, i.e., *P. contorta* growing in its native or introduced range) and “native-introduced region pair” (NIRP, i.e., Swedish *P. contorta* subsp. *latifolia* originating from Canada or Patagonian *P. contorta* subsp. *murrayana* originating from the USA) as two fixed factors were used to assess variations in community composition. Due to the non-normality and heteroscedasticity of the richness and relative abundance data across *P. contorta* in native and introduced regions, Kruskal-Wallis tests were conducted with the aforementioned fixed factors. Similarly, for our second hypothesis, one-factor PERMANOVAs were employed to compare the community composition differences between *P. contorta* subsp. *murrayana* plantations and their paired invasion fronts in Patagonia, and homogeneity of multivariate dispersion tests were conducted to assess within-group uniformity. A Wilcoxon rank sum test was employed to compare the richness of the “others” guild between the plantations and invasion fronts in Patagonia, given the non-normality and heteroscedasticity of the data. The richness of all categories, excluding the “others” guild, and the relative abundance of all guilds were examined using Welch’s t-tests. Principal coordinate analysis based on Bray-Curtis distance (PCoA, “cmdscale” function in “vegan” package) was employed to visually represent multivariate comparisons. For the plant pathogens, we conducted multivariate comparisons based on Bray-Curtis and Jaccard distances, which rely on species relative abundance and presence/absence, respectively. All distance matrices were constructed using the “vegdist” function in the “vegan” package, and species-relative abundance data were used after normalization. Kruskal-Wallis tests were conducted using the “scheirerRayHare” function from the “rcompanion” package. In cases where significant interaction effects were detected (α = 0.05), we followed up with pairwise Wilcoxon rank sum tests, employing Holm-Bonferroni adjusted *p*-values for multiple comparisons. Further, we conducted a “contribution of variables to similarity (SIMPER)” analysis using the “simper” function within the “vegan” package. This analysis aimed to identify the fungal species that primarily contributed to the similarity within groups as well as the dissimilarity among groups.

## Results

After adapter removal and quality control, a total of 6,259 unique ASVs across all sites and stand types were obtained. The number of ASVs in each category for each region and tree type is reported in Table S1. The ASVs that contributed the most to the similarity within each of these groups are reported in Table S2.

### *Pinus contorta* fungal communities across different regions

**Fig. 2:**
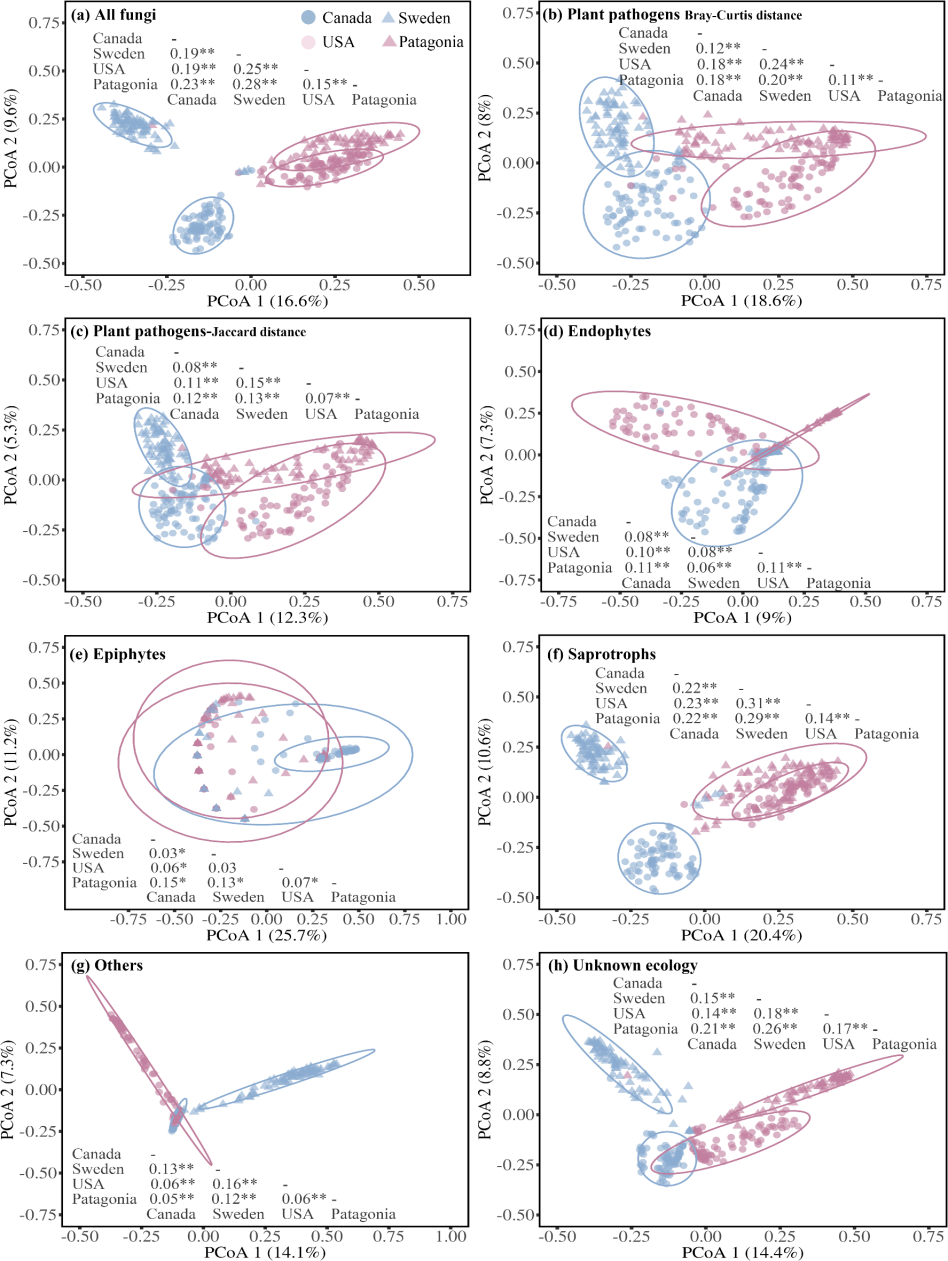
A principal coordinate analysis (PCoA) showing the results of a PERMANOVA test evaluating differences in community composition of (**a**) all fungi, (**b**,** c**) plant pathogens, (**d**) endophytes, (**e**) epiphytes, (**f**) saprotrophs, (**g**) “others” and (**h**) fungi with unknown ecology associated with *Pinus contorta* needles in four distinct regions (Canada, USA, Sweden, and Patagonia). Results of the PERMANOVA are reported in Table 1. The R^2^ of post-hoc pairwise comparisons are shown on each panel, number with *, and ** indicates a significant difference at α = 0.05 and α = 0.01 respectively.

Community composition of all fungal categories associated with *P. contorta* was responsive to the main effects of introduction status (IS; *P. contorta* in its native vs. introduced range), native-introduced region pairs (NIRPs; northern NIRP, Canada—Sweden; or southern NIRP, USA—Patagonia), and their interactions (Table 1; Fig. 2). The differences in fungal community dissimilarity among the northern and the southern NIRP regions led to interaction effects between IS and NIRP. For all fungi, plant pathogens, saprotrophs, and “others”, the interaction effects among IS and NIRP arose because communities across the southern NIRP were more similar than the northern NIRP (Fig. 2a,b,c,f,g). For endophytes, epiphytes, and unknown ecology, the interaction effects occurred because the communities across the northern NIRP were more similar than the southern NIRP (Fig. 2d,e,h). Additionally, the multivariate dispersion of each fungal category across native and introduced regions was significantly different, with the average distance to the median ranging from 0.45 to 0.68. Notably, in the northern NIRP, the dispersion of all fungal categories, except for endophytes, was significantly higher in Canada as compared to Sweden, whereas the pattern in the southern NIRP was less pronounced (Fig. S1).

For the northern NIRP, ASVs assigned to *Lophodermium baculiferum*, *Phaeotheca fissurella*, *Lophodermella concolor*, *Perusta inaequalis*, and *Cladosporium basiinflatum* were the dominant plant pathogens contributing the most to the dissimilarity between *P. contorta* subsp. *latifolia* in Sweden and Canada (Table S3). The relative abundance of these pathogens was generally higher in Canada than in Sweden, except *P. inaequalis*, which exhibited a marginally higher abundance in Sweden (Table S3). For the southern NIRP, similarly, ASVs assigned to *P. fissurella*, *L. baculiferum*, as well as *Lophodermium pinastri*, *Meristemomyces arctostaphyli*, and *Neophaeomoniella constricta* were the predominant plant pathogens that contributed the most to the dissimilarity between *P. contorta* subsp. *murrayana* in Patagonia and the USA (Table S4). Among these, *P. fissurella*, *L. baculiferum*, and *M. arctostaphyli* had higher relative abundance in the USA, whereas *L. pinastri* and *N. constricta* were more abundant in Patagonia (Table S4).

**Fig. 3:**
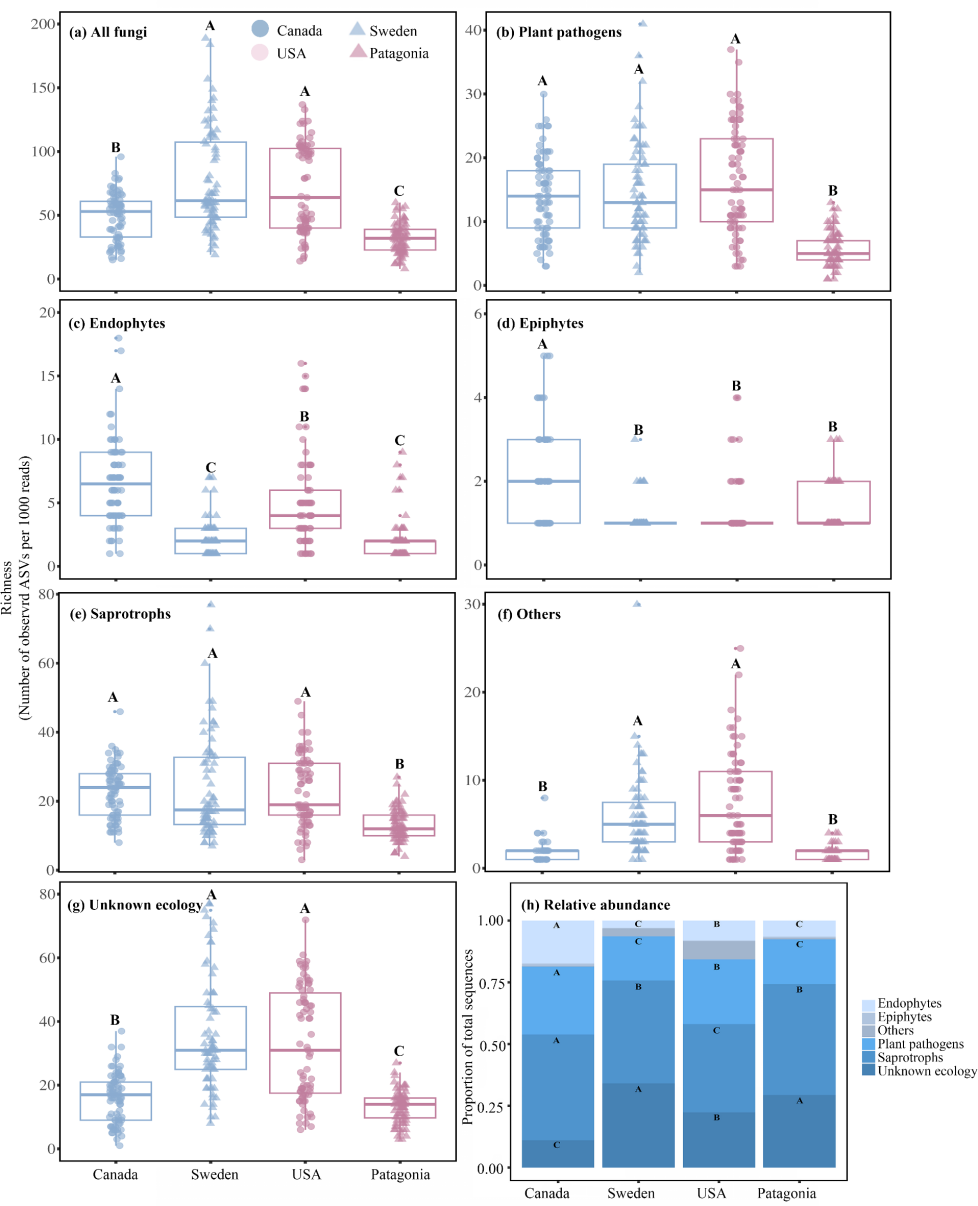
Amplicon sequence variants (ASVs) richness (**a**–**g**) and relative abundance (**h**) of fungal communities associated with *Pinus contorta* needles in two native range populations (Canada and USA) and two introduced populations (Sweden and Patagonia), respectively. Richness is presented as the average rarified richness for each sample. Results from the corresponding two-way Kruskal-Wallis test for all variables are reported in Table 1. Different letters above boxplots (**a**–**g**) or across bar segments with the same shade (**h**) indicate significant pairwise differences (α = 0.05) determined using nonparametric post hoc comparisons.

For richness and relative abundance variables, introduction status (IS) significantly affected the richness of all fungi, plant pathogens, endophytes, epiphytes, and saprotrophs, as well as the relative abundance of plant pathogens, endophytes, epiphytes, saprotrophs, and unknown ecology (Table 1). The native-introduced region pair (NIRP) significantly affected the richness of total fungi, plant pathogens, endophytes, epiphytes, saprotrophs, and unknown ecology, as well as the relative abundance of endophytes, saprotrophs, “others”, and unknown ecology (Table 1). Further, the richness of all fungi, plant pathogens, epiphytes, saprotrophs, “others”, and unknown ecology, as well as the relative abundance of endophytes, epiphytes, saprotrophs, “others”, and unknown ecology were affected by the interaction of IS and NIRP (Table 1). Within the northern NIRP, the richness of all fungi, “others”, and unknown ecology was higher in the introduced relative to native range (Fig. 3a,f,g), whereas the opposite was observed in endophytes and epiphytes (Fig. 3c,d). However, no significant differences in the richness of plant pathogens and saprotrophs were detected between the two regions (Fig. 3b,e). In the southern NIRP, a lower richness of all categories except epiphytes was detected in the introduced relative to the native range (Fig. 3).

For the relative abundance data, plant pathogens and endophytes of *P. contorta* plantations comprised a greater proportion in the native as compared to the introduced regions, while the opposite occurred for the unknown ecology group (Fig. 3h). For saprotrophs, they had a greater relative abundance of the fungal community in the native than in the introduced regions in the northern NIRP (i.e., higher in Canada than Sweden) while the opposite was observed in the southern NIRP (i.e., higher in Patagonia than the USA) (Fig. 3h).

**Table 1:** The results from two-factor PERMANOVA (F) or Kruskal-Wallis (H) tests evaluating *Pinus contorta* needle fungal responses to introduction status (i.e., growing in its native vs. introduced range) and between two different native-introduced region pairs (populations from Canada or USA introduced to Sweden or Patagonia, respectively). Analysis of community composition was done using PERMANOVA on a Bray-Curtis similarity matrix of fungal taxonomic units identified using PacBio, while richness and relative abundance were analyzed using Kruskal-Wallis tests.

	Introduction status (IS)				Native-introduced region pair (NIRP)				IS×NIRP		
	F or H		*p* value		F or H		*p* value		F or H		*p* value
Community composition											
All fungi	32.33		**0.001**		60.30		**0.001**		29.80		**0.001**
Plant pathogens	23.13		**0.001**		55.20		**0.001**		15.89		**0.001**
Endophytes	10.34		**0.001**		8.59		**0.001**		7.78		**0.001**
Epiphytes	4.97		**0.001**		18.84		**0.001**		6.29		**0.001**
Saprotrophs	34.69		**0.001**		75.07		**0.001**		30.26		**0.001**
Others	10.17		**0.001**		19.14		**0.001**		10.18		**0.001**
Unknown ecology	28.79		**0.001**		43.74		**0.001**		27.79		**0.001**
Richness											
All fungi	8.35		**0.003**		19.48		**< 0.001**		67.79		**< 0.001**
Plant pathogens	38.63		**< 0.001**		21.25		**< 0.001**		41.33		**< 0.001**
Endophytes	90.97		**< 0.001**		7.64		**0.006**		2.57		0.109
Epiphytes	8.63		**0.003**		6.36		**0.012**		10.27		**0.001**
Saprotrophs	30.89		**< 0.001**		19.52		**< 0.001**		13.87		**< 0.001**
Others	0.58		0.446		0.17		0.679		78.82		**< 0.001**
Unknown ecology	0.28		0.595		6.33		**0.012**		111.00		**< 0.001**
Relative abundance											
Plant pathogens	99.29		**< 0.001**		1.68		0.195		1.97		0.161
Endophytes	93.23		**< 0.001**		14.03		**< 0.001**		11.70		**< 0.001**
Epiphytes	9.01		**0.003**		0.18		0.670		30.85		**< 0.001**
Saprotrophs	18.75		**< 0.001**		5.74		**0.017**		41.69		**< 0.001**
Others	3.50		0.062		6.36		**0.013**		179.10		**< 0.001**
Unknown ecology	50.80		**< 0.001**		11.62		**< 0.001**		22.15		**< 0.001**

For community composition data, the F-value is generated using permutation procedures and thus is referred to as a Pseudo-F-value. For richness and relative abundance, Kruskal-Wallis H values are used. *p* values in bold are significant at α = 0.05.

### Fungal communities of introduced *Pinus contorta* subsp. *murrayana* plantations vs. invasion fronts in Patagonia

**Fig. 4:**
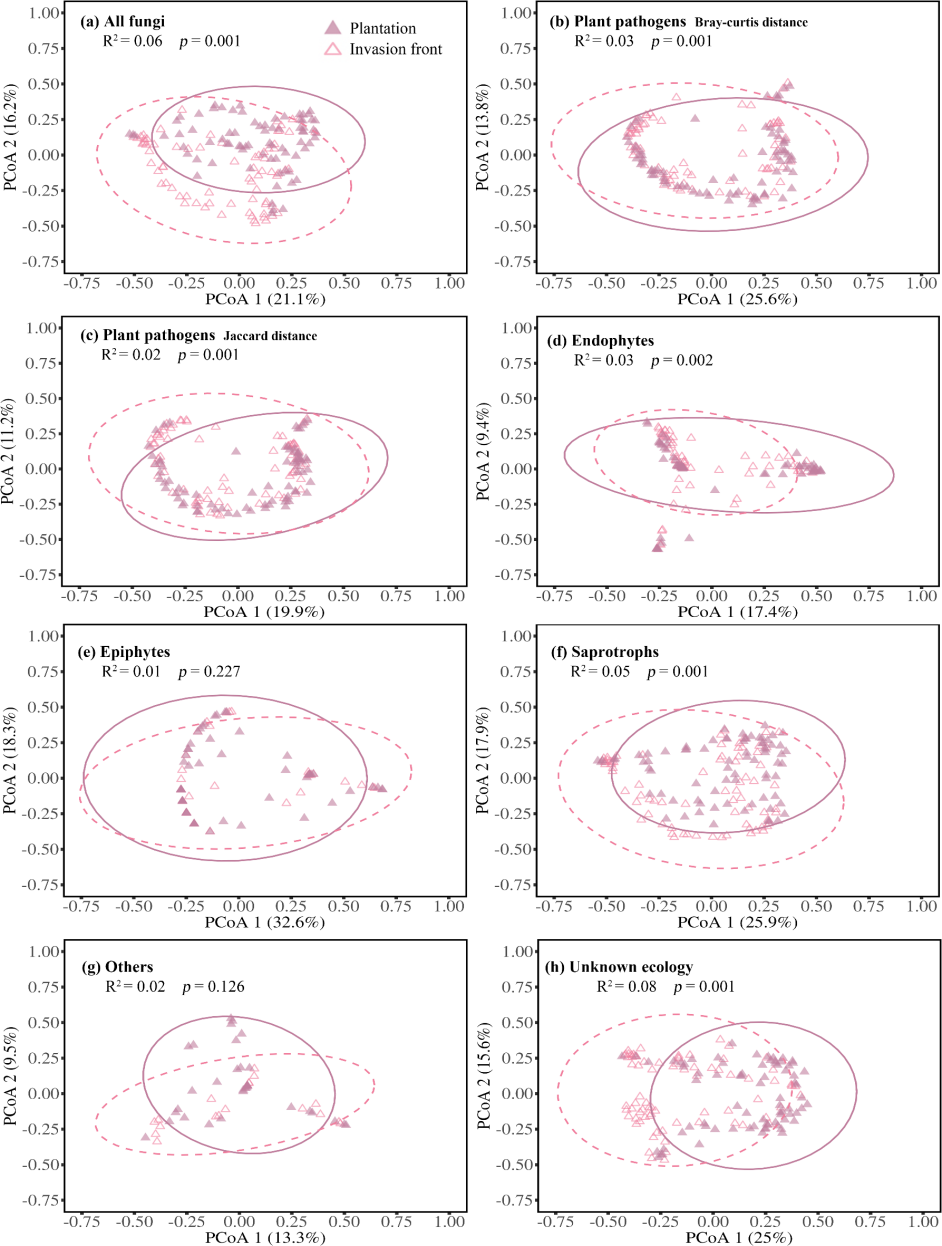
A principal coordinate analysis (PCoA) showing the results of a PERMANOVA test evaluating differences in community composition of (**a**) all fungi, (**b**,** c**) plant pathogens, (**d**) endophytes, (**e**) epiphytes, (**f**) saprotrophs, (**g**) “others” and (**h**) fungi with unknown ecology associated with *Pinus contorta* needles in introduced plantations and invasion fronts growing from plantations in Patagonia. Results of the PERMANOVA are reported in Table S5, with α = 0.05 indicating statistically significant differences.

All fungal community composition variables, except epiphytes and “others”, significantly differed between Patagonia plantations and adjacent invasion fronts (Table S5; Fig. 4). Differences in plant pathogen communities were observed irrespective of whether Bray-Curtis or Jaccard distance metrics were applied (Fig. 4b,c). Additionally, a significantly greater multivariate dispersion of unknown ecology was detected within invasion fronts compared to plantations, with average distances to the median of 0.56 and 0.49, respectively (W = 16,537,411, *p* = 0.004, Wilcoxon rank sum test). The total dissimilarity between *P. contorta* subsp. *murrayana* plantations and invasion fronts in Patagonia was 78.2%. Among plant pathogens, *P. fissurella*, *L. pinastri*, and *N. constricta* contributed the most to the dissimilarity (Table S6). Specifically, the abundance of *P. fissurella* was greater in the invasion front compared to the plantations, whereas the abundance of *L. pinastri* and *N. constricta* was similar in both the plantations and the invasion fronts (Table S6).

**Fig. 5:**
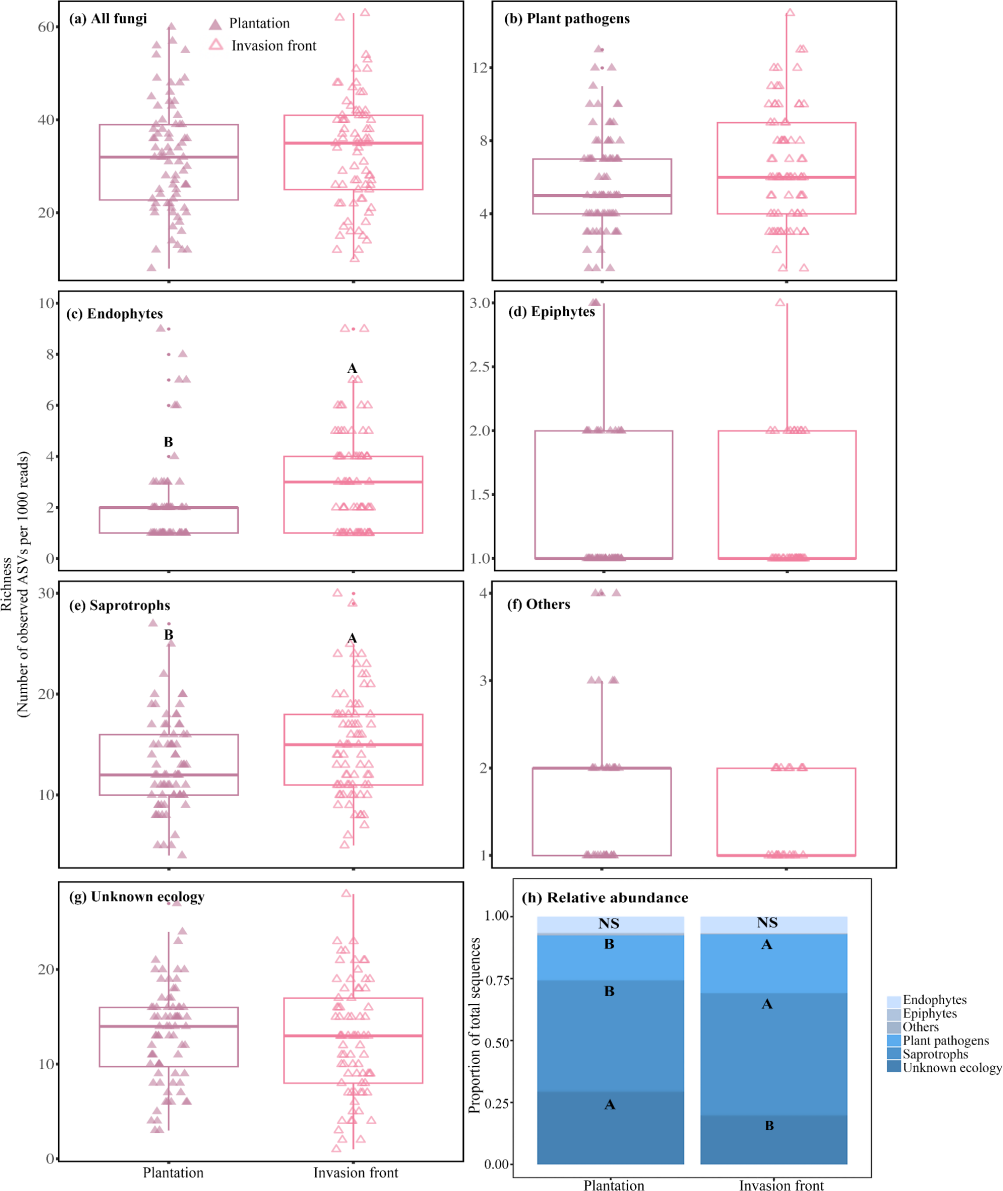
Amplicon sequence variants (ASVs) richness (**a**–**g**) and relative abundance (**h**) of fungal communities associated with *Pinus contorta* subsp. *murrayana* needles in introduced plantations and invasion fronts growing from plantations in Patagonia, respectively. Richness is presented as the average rarified richness for each sample. Results from the corresponding Welch’s t or Wilcoxon rank sum tests are reported in Table S5. Different letters above boxplots (**a**–**g**) or across bar segments with the same shade (**h**) indicate significant differences at α = 0.05.

The richness of endophytes and saprotrophs was significantly greater in invasion fronts as compared to plantations (Table S5; Fig. 5c,e). For the relative abundance data, significant differences were observed across all fungi and other categories, except endophytes (Table S5). The relative abundance of plant pathogens and saprotrophs was higher in *P. contorta* subsp. *murrayana* invasion fronts compared to plantations, whereas fungi with unknown ecology exhibited a lower relative abundance in the invasion fronts relative to plantations (Fig. 5h).

## Discussion

We observed significant differences in foliar fungal composition, species richness, and relative abundance following the large-scale introduction of *P. contorta* from North America to both Sweden and Patagonia. Additionally, we identified substantial variation in the foliar fungal community between invasion fronts and source plantations within Patagonia. We now discuss these patterns in relation to our initial hypotheses.

### Foliar fungal communities of *Pinus contorta* in its native versus introduced ranges

Exotic species interact with local ecosystems through mechanisms such as microbial co-evolution and adaptation, leading to shifts in the community composition of both introduced and native species within new environments^53^. In the context of *P. contorta* introduction from North America to Sweden and Patagonia, we predicted that *P. contorta* would be associated with distinct and less species-rich foliar fungal communities in its introduced compared to native range (H1a). In support of our first hypothesis, we observed a significant alteration of the foliar fungal community composition across all native and introduced populations (Fig. 2). Additionally, the relative abundance of plant pathogens on average was significantly reduced in the introduced compared to native regions (Fig. 3h), and we found a significantly decreased richness of pathogens, endophytes, epiphytes, and saprotrophs in the introduced regions, as compared to native regions (Fig. S2). These findings indicate that in general *P. contorta* has undergone foliar fungal community composition shifts, including pathogen release following large-scale introductions, which could be one of several factors contributing to its enhanced growth or successful invasion in its introduced areas^21,26^.

### Contrasting Northern versus Southern native-introduced region pairs

The presence or absence of phylogenetically related native species in areas of introduction has the potential to influence various ecological processes that shape associated fungal community composition^54^. For instance, within our study system, Gundale, et al.^18^ observed that soil fungal community composition was less similar between *P. contorta* plantations in the United States and the Southern Hemisphere, where phylogenetically related tree species are absent, compared to populations in Canada and Europe. Given that foliage is more susceptible to airborne fungal spores from the surrounding environment and considering the presence of the phylogenetically related *P. sylvestris* in Sweden, we predicted that differences in the *P. contorta* foliar fungal community between native and introduced ranges would be more pronounced in the southern compared to the northern NIRP (H1b). We found that the composition, richness, and relative abundance of nearly all fungal categories were significantly affected by native-introduced region pair (NIRP; Table 1; Figs. 2 and 3).

In the northern NIRP, the greater influx of local, wind-dispersed fungal spores from the phylogenetically close *P. sylvestris* into the *P. contorta* plantations in Sweden^14,34^ may explain the distinctly different foliar fungal communities between Sweden and Canada (Figs. 2 and 3). In support of this, several generalist species such as *Phaeotheca* sp.^55^, *Lophodermium conigenum*^56^, and *Hyphodiscus* sp.^57^, sequenced from Swedish *P. contorta* needles are species well known to occur in European *P. sylvestris* forests, but were absent from the Canadian sites examined in our study (Tables S2 and S3). These local fungal taxa, as well as widely distributed generalists at Swedish sites, could also exert priority effects that impede the establishment of the common Canadian taxa^20,21^.

For plant pathogens, although the overall richness did not differ significantly between Canada and Sweden (Fig. 3b), we found a significantly lower relative abundance of plant pathogens in Swedish plantations compared to Canadian stands (Fig. 3h). Further, several prominent plant pathogens were present in Canadian sites but absent in Swedish sites, including *L. baculiferum*, *L. concolor*, and *L. resinosum* (Tables S2 andS3). These taxa are primarily distributed or originate in North America^58,59^, and their absence at our Swedish sites offers robust support for ERH in the northern NIRP pair^60,61^. Furthermore, the generalist plant pathogen *Neophaeomoniella constricta* was recently identified as a new taxon^62^, and in our study, this species existed both in Sweden and Canada but had a higher abundance in Swedish *P. contorta* sites compared to Canadian counterparts (Table S3). Taken together, *P. contorta* underwent significant foliar fungal community changes, including release from some specific pathogens, during its introduction across the northern NIRP, with the literature indicating that communities commonly associated with phylogenetically close *P. sylvestris* in Sweden influenced the fungal communities of *P. contorta*.

In the southern NIRP, the altered foliar fungal community composition (Fig. 2) and the significant reduction in foliar fungal richness across all fungal categories, except for epiphytes, in *P. contorta* plantations in Patagonia, compared to those in the USA (Fig. 3), indicate that the introduction process has caused large changes in the fungal community. Moreover, the observed decrease in both the richness and relative abundance of plant pathogens in Patagonia compared to the USA (Fig. 3b,h) indicates that pathogen release occurred during *P. contorta*’s introduction from the USA to Patagonia, and the co-invasion of fungal taxa from the USA to Patagonia is a primary assembly mechanism for Patagonian foliar fungal communities^19^. In support of this, we identified several crucial plant pathogens, including the pathogens *P. fissurella* and *L. baculiferum*, both of which were found to be more abundant in the USA than in Patagonia (Table S4). Notably, the literature indicates these pathogens are native to coniferous forests in the Northern Hemisphere and are absent from the *Nothofagus* forests of the Southern Hemisphere^58,63^, indicating that they have followed a co-introduction trajectory into Patagonian *P. contorta* plantations. These data suggest that relatively few taxa from local Patagonian *Nothofagus* forests, with notable exceptions such as the generalist saprotroph *Hormonema macrosporum*^64^, can colonize *P. contorta*, and that co-invasion from the Northern Hemisphere is a key process that has led to significant changes in community composition, reduced species richness, and pathogen release following the introduction of *P. contorta* from the USA to Patagonia. The local randomness of co-invasion may also explain why pathogens in Patagonia exhibit higher beta-dispersion than the native range (Fig. S1), which could occur if different co-invading taxa initially dominate at different sites.

Taken together, we observed significant differences in foliar fungal community composition, along with reduced richness and relative abundance of plant pathogens, following the introduction of *P. contorta* across a broad geographic range—particularly in the southern NIRP (Figs. 3 and S2; Tables S2, S3, and S4). Consequently, our first hypothesis was supported that *P. contorta* would associate with distinct foliar fungal communities and undergo pathogen release during its large-scale introduction. The reduction in fungal richness appeared to be especially pronounced in Patagonia (Fig. 3), where phylogenetically related tree species are absent, whereas changes in composition appeared to be strongest in the northern NIRP (Fig. 2), where a phylogenetically similar tree species was present. These findings suggest that distinct ecological processes operated in introduction contexts with varying phylogenetic similarity. In Sweden, the presence of the phylogenetically related *P. sylvestris* appears to significantly influence the colonization and composition of foliar fungal communities in *P. contorta* plantations. The relatively greater dissimilarity between Swedish and Canadian *P. contorta* fungal communities can thus plausibly be explained by a distinct yet partially suitable foliar fungal community associated with *P. sylvestris* in Sweden. In contrast, in Patagonia, the partial co-invasion of fungal taxa accompanying *P. contorta* from North America appears to play a role, albeit gradually, in reducing fungal community richness and abundance, thereby facilitating pathogen release within the southern NIRP. Thus, community assembly via co-invasion in Patagonia may help explain the relatively greater community similarity in the southern (93.3%) than northern (87.9%) NIRP, the strong reduction in richness (Fig. S2), and greater pathogen community dispersion in Patagonia compared to North America (Fig. S1).

### Foliar fungal communities of Patagonian plantations versus invaders

At the local scale, microbe-climate differences between source plantations and invasion fronts can lead to fungal dispersal limitations, which subsequently influence microbial co-invasion and invasion success; however, as the invasion progresses, micro-environments are likely to converge^65^. For example, in the same stand network, Núñez, et al.^39^ demonstrated that the key belowground fungal mutualists were restricted to areas close to source plantations, while lower levels of mycorrhizal colonization and fewer fungal species were observed at greater distances from the original plantings. In our study, the presence of common fungal species across plantations and invasion fronts (Table S6), along with the lack of significant differences in multivariate dispersion across nearly all fungal categories, suggests that dispersal limitation is not a major factor influencing foliar fungal communities over distances spanning from plantations to invasion fronts. Although the differences in fungal composition between source plantations and invasion fronts were relatively modest (R² ranging from 0.01 to 0.06 for all fungi, pathogens, endophytes, saprotrophs, and unknown ecological groups; Fig. 4), and fungal richness differences were also limited (Fig. 5), our results suggest that fungal community divergence was primarily driven by differences in species relative abundances rather than species presence.

Factors such as differences in tree age or degree of canopy heterogeneity may lead to distinct microenvironments that influence the relative abundances of fungal taxa^25,66,67^. This pattern, consistent with Gundale, et al.^18^, contrasts the larger inter-continental scale comparison we made between native stands in the USA and Patagonia plantations (Fig. 3; Tables S2 andS4), where differences in species presence-absence were a larger contributor to the difference in community composition, which corresponded with a reduction in richness in Patagonia. In brief, while significant alterations in fungal community composition were observed between *P. contorta* plantations and invasion fronts in Patagonia, there was insufficient evidence to suggest dispersal limitation or pathogen release occurs at this scale during the spread of *P. contorta* from source plantations.

To date, no studies have investigated the variation in foliar fungal communities of *P. contorta* across such an extensive geographical range, incorporating replicated native and introduced sites along with the phylogenetic similarity to the resident plant community. Our study reveals that assembly mechanisms shaping foliar fungal communities differ significantly across various environmental contexts and also highlights the critical role that foliar fungal communities can have in intercontinental introductions, where pathogen release can contribute to a species’ establishment and invasion success.

## Electronic supplementary material

Below is the link to the electronic supplementary material.

Supplementary Material 1

Supplementary Material 2

## Data Availability

The datasets generated and/or analyzed during the current study are available in the European Nucleotide Archive (ENA), accession number is PRJEB82887.
