# Supplementary material for: Distinct foliar fungal communities in Pinus contorta across native and introduced ranges: evidence for context dependency of pathogen release
Source: Sci Rep. 2025 Mar 1;15:7273. doi: 10.1038/s41598-025-91639-z (PMC11873135; doi:10.1038/s41598-025-91639-z)
Supplement: Supplementary file 1 — Supplementary Material 1 [file 41598_2025_91639_MOESM1_ESM.docx]

**Supplementary Material**

**Table S1.** The cumulative number of plant pathogens, endophytes, epiphytes, saprotrophs, others, unknown ecology, and total amplicon sequence variants (ASVs) found across all stands of a given tree type within a specific region.

| Region | Introduction status | Plant pathogens | Endophytes | Epiphytes | Saprotrophs | Others | Unknown ecology | Total |
| --- | --- | --- | --- | --- | --- | --- | --- | --- |
| Canada | native-plantation | 323 | 152 | 39 | 509 | 42 | 436 | 1501 |
| Sweden | introduced-plantation | 438 | 88 | 25 | 781 | 197 | 1150 | 2679 |
| USA | introduced-plantation | 434 | 142 | 31 | 651 | 323 | 1259 | 2840 |
| Patagonia | introduced-plantation | 96 | 36 | 13 | 201 | 33 | 213 | 592 |
|  | invasion front | 163 | 65 | 18 | 313 | 13 | 299 | 871 |

**Table S2**: A list of fungal amplicon sequence variants (ASVs) and their functional classification contributing most to the similarity within each of 5 tree groups, including *P. contorta* subsp. *latifolia* stands in Canada and Sweden, *P. contorta* subsp. *murrayana* in the USA and Patagonia, and invasion fronts growing from *P. contorta* subsp. *murrayana* plantations in Patagonia. We only list species contributing to the upper 70% of within-group similarity.

| ***P. cintorta* subsp.**  ***latifolia* , Canada (79.57%)** | | |  |  | | |  |  |  |
| --- | --- | --- | --- | --- | --- | --- | --- | --- | --- |
| ASV | Species | Functional classification | | | Average abundance | | Average similarity (%) | Similarity contribution (%) | Cumulative contribution (%) |
| 31dece8c6d61e8794e2dd50737d339d3 | *Hormonema macrosporum* | Undefined Saprotroph | | | 0.09 | | 4.84 | 6.08 | 6.08 |
| 77a3857ab7c279cf4dcfbd0a9cc4880c | *Phaeococcomyces mexicanus* | Undefined Saprotroph | | | 0.07 | | 3.44 | 4.32 | 10.40 |
| 1d0e5bdf9d3bde620f87966848cd97d5 | *Lophodermium baculiferum* | Endophyte-Leaf Saprotroph-Plant Pathogen | | | 0.04 | | 2.99 | 3.76 | 14.16 |
| ed6643c22af17ce8a5a199b3938a3d54 | *Lophodermella concolor* | Endophyte-Leaf Saprotroph-Plant Pathogen | | | 0.03 | | 2.83 | 3.55 | 17.71 |
| 289f6cd604d766d3681f35c0c9b2223f | *Phaeotheca fissurella* | Plant Pathogen-Plant Saprotroph | | | 0.04 | | 2.40 | 3.01 | 20.73 |
| 8cbafb6aca8ca31b83c00f6928e2c79c | *Lophodermium baculiferum* | Endophyte-Leaf Saprotroph-Plant Pathogen | | | 0.03 | | 2.24 | 2.81 | 23.54 |
| c65f504e999765f2ce42ad83da5de55e | *Lophodermium baculiferum* | Endophyte-Leaf Saprotroph-Plant Pathogen | | | 0.03 | | 1.92 | 2.41 | 25.95 |
| 06fdd6a4c255731826571c830e379bb3 | *Lophodermium baculiferum* | Endophyte-Leaf Saprotroph-Plant Pathogen | | | 0.02 | | 1.72 | 2.16 | 28.11 |
| ae387a169b00b249d56547ee9d9fd350 | Mrakiaceae sp | Unknown | | | 0.02 | | 1.62 | 2.03 | 30.14 |
| ee7ce27261257e5115a91c70ba994dec | *Hormonema macrosporum* | Undefined Saprotroph | | | 0.02 | | 1.49 | 1.88 | 32.02 |
| 5b9c21feabf050582a54f3226c34805b | *Cladosporium basi-inflatum* | Animal Pathogen-Endophyte-Lichen Parasite-Plant Pathogen-Wood Saprotroph | | | 0.02 | | 1.47 | 1.85 | 33.86 |
| 35f1cedf646ad1ff5d15caf5fe097494 | *Phaeotheca fissurella* | Plant Pathogen-Plant Saprotroph | | | 0.02 | | 1.29 | 1.62 | 35.49 |
| e08503cf4f976fa7f12fd6be62895312 | *Lapidomyces hispanicus* | Animal Pathogen-Plant Pathogen-Undefined Saprotroph | | | 0.02 | | 1.25 | 1.57 | 37.06 |
| 142925e4aea7cfecc2d457dc28e9481f | *Lophodermium baculiferum* | Endophyte-Leaf Saprotroph-Plant Pathogen | | | 0.01 | | 1.02 | 1.28 | 38.34 |
| d23c794d2e3517c216ec1025795aef4a | *Perusta inaequalis* | Undefined Saprotroph | | | 0.02 | | 1.00 | 1.25 | 39.59 |
| 3903170797690df40698e22775f918f9 | Myriangiales sp | Unknown | | | 0.01 | | 0.91 | 1.15 | 40.73 |
| fcce34aef79c07cddb8c73bbbb2af57a | *Phaeotheca fissurella* | Plant Pathogen-Plant Saprotroph | | | 0.01 | | 0.84 | 1.06 | 41.79 |
| 9df0cee9028d725c973459e4761c4d72 | Unclassified | Unknown | | | 0.01 | | 0.79 | 0.99 | 42.78 |
| 7f3aa1241c8212141df9a7400c4101da | Unclassified | Unknown | | | 0.01 | | 0.78 | 0.98 | 43.76 |
| e4e4a368868f4ba65b9b0f6c18185c83 | Myriangiales sp | Unknown | | | 0.01 | | 0.69 | 0.87 | 44.63 |
| 9c9b7bc54bf6cd74f9547eaf2fa4b39c | *Lapidomyces hispanicus* | Animal Pathogen-Plant Pathogen-Undefined Saprotroph | | | 0.01 | | 0.66 | 0.83 | 45.46 |
| 0d96521fc9e17f316bff93e54778c2ce | Unclassified | Animal Pathogen-Plant Pathogen-Undefined Saprotroph | | | 0.01 | | 0.64 | 0.81 | 46.27 |
| 26e6e088ab5645b96e3698d5102a4257 | *Lophodermium baculiferum* | Endophyte-Leaf Saprotroph-Plant Pathogen | | | 0.01 | | 0.63 | 0.79 | 47.06 |
| 891aaffd033d6ab6fc788c6d0800f284 | Unclassified | Plant Pathogen-Undefined Saprotroph | | | 0.01 | | 0.62 | 0.78 | 47.85 |
| 7e7e3651ca5b16042b98e03c56eeb79b | *Neophaeomoniella constricta* | Plant Pathogen | | | 0.01 | | 0.58 | 0.73 | 48.58 |
| b1b64ea48ee82b6f4b3137339dbe8cd5 | *Phaeococcomyces mexicanus* | Undefined Saprotroph | | | 0.01 | | 0.56 | 0.71 | 49.28 |
| 28759794967a7c39dcf158c8409215be | *Hormonema macrosporum* | Undefined Saprotroph | | | 0.01 | | 0.56 | 0.71 | 49.99 |
| ded2771c98ec82002e74d2ab16f661bc | *Lophodermium baculiferum* | Endophyte-Leaf Saprotroph-Plant Pathogen | | | 0.01 | | 0.55 | 0.70 | 50.68 |
| 12a4c9f2c63f0bf7d366431144d21278 | Unclassified | Unknown | | | 0.01 | | 0.54 | 0.68 | 51.36 |
| 931d5d984a4c9edd64e4b9eeef39df5e | Pseudeurotiaceae sp | Plant Saprotroph-Wood Saprotroph | | | 0.01 | | 0.53 | 0.67 | 52.03 |
| 3307132c681cc78f616436f2ea32743a | *Lophodermium resinosum* | Endophyte-Leaf Saprotroph-Plant Pathogen | | | 0.01 | | 0.52 | 0.65 | 52.68 |
| 2bae77c3c87daa6d002730d61da23d34 | *Allantophomopsiella pseudotsugae* | Plant Pathogen | | | 0.00 | | 0.50 | 0.62 | 53.30 |
| 4fe20330dff299e451c55745c12c7f74 | Capnocheirides sp | Animal Pathogen-Plant Pathogen-Undefined Saprotroph | | | 0.01 | | 0.49 | 0.61 | 53.91 |
| d1d39b676469dd14f6e83bbefbc6235a | Unclassified | Unknown | | | 0.01 | | 0.49 | 0.61 | 54.53 |
| aff8b0eb211ef9900fbf9067b156fecf | Unclassified | Endophyte-Leaf Saprotroph-Plant Pathogen | | | 0.00 | | 0.47 | 0.60 | 55.12 |
| f93af6ffe0060096694b89af1bb46596 | *Lophodermium baculiferum* | Endophyte-Leaf Saprotroph-Plant Pathogen | | | 0.00 | | 0.46 | 0.57 | 55.69 |
| 491231c0fb35030bb280feb5c3a8db2f | Unclassified | Epiphyte | | | 0.01 | | 0.45 | 0.57 | 56.27 |
| b251ad15a9ad7f44b68a8ddaa6bcc93d | *Perusta inaequalis* | Undefined Saprotroph | | | 0.01 | | 0.44 | 0.55 | 56.82 |
| b20e3ec8817c42fe8a7fa978962add39 | *Genolevuria tibetensis* | Fungal Parasite-Undefined Saprotroph | | | 0.01 | | 0.42 | 0.53 | 57.35 |
| b3fc0f62ae7fe0ee6e1d884ebe0fbdb3 | Phaeothecaceae sp | Unknown | | | 0.01 | | 0.41 | 0.51 | 57.86 |
| 7c6f38afb12b00357089d887d03261d3 | *Lophodermium baculiferum* | Endophyte-Leaf Saprotroph-Plant Pathogen | | | 0.00 | | 0.39 | 0.49 | 58.35 |
| b00637bbb6ec4862e48e22bb19e934e6 | Unclassified | Plant Pathogen | | | 0.00 | | 0.39 | 0.49 | 58.84 |
| a79fd5e4353832dc646678cfbb16ce91 | Unclassified | Unknown | | | 0.00 | | 0.39 | 0.49 | 59.33 |
| 2ba99bc7bf0258de782849a61eaf7a3f | *Lophodermium molitoris* | Endophyte-Leaf Saprotroph-Plant Pathogen | | | 0.00 | | 0.38 | 0.48 | 59.81 |
| 54e9cfcbbe8e4cdaf40a61f162c22827 | *Phaeotheca fissurella* | Plant Pathogen-Plant Saprotroph | | | 0.00 | | 0.36 | 0.46 | 60.26 |
| eb88a644353f4e6ebd8fd9600bd90e21 | Perusta sp | Undefined Saprotroph | | | 0.00 | | 0.35 | 0.44 | 60.71 |
| 58dc2388bdf13a3259088459c94d1719 | *Hormonema macrosporum* | Undefined Saprotroph | | | 0.00 | | 0.35 | 0.43 | 61.14 |
| b437379ba9e1476557cc66544d91c5f0 | Unclassified | Endophyte-Plant Pathogen-Undefined Saprotroph | | | 0.00 | | 0.34 | 0.43 | 61.57 |
| 7e4f4afdcbb411b247a341ca5df104fc | Fungi sp | Unknown | | | 0.00 | | 0.34 | 0.43 | 62.00 |
| c868ff643495266126e7d26733e7b4bd | *Lophodermium molitoris* | Endophyte-Leaf Saprotroph-Plant Pathogen | | | 0.00 | | 0.34 | 0.43 | 62.44 |
| 744e3313691ebbac20552e3b64401c14 | *Lophodermium resinosum* | Endophyte-Leaf Saprotroph-Plant Pathogen | | | 0.00 | | 0.33 | 0.41 | 62.85 |
| 80a9f4f9b3c47f908ad4cb2fe9221239 | *Lophodermella montivaga* | Endophyte-Leaf Saprotroph-Plant Pathogen | | | 0.00 | | 0.31 | 0.39 | 63.24 |
| 04d4624979dff0f162d0cb536c33ed1e | *Lophodermium resinosum* | Endophyte-Leaf Saprotroph-Plant Pathogen | | | 0.00 | | 0.30 | 0.38 | 63.62 |
| e7de469d4e75395faec40320701a7c39 | *Sarcinomyces crustaceus* | Endophyte | | | 0.00 | | 0.30 | 0.37 | 63.99 |
| 8886792f31e4ed4947ea99bcff9aa922 | Unclassified | Unknown | | | 0.00 | | 0.29 | 0.36 | 64.35 |
| 3f546a9c02ff60c22e099cf989bbd9e6 | Unclassified | Endophyte-Leaf Saprotroph-Plant Pathogen | | | 0.00 | | 0.26 | 0.33 | 64.68 |
| bf3e663da7f2ee038bd174c5f06e62c2 | *Lophodermium baculiferum* | Endophyte-Leaf Saprotroph-Plant Pathogen | | | 0.00 | | 0.26 | 0.32 | 65.00 |
| a5d23f85a3113a04e48974883d153b77 | *Amphosoma atroolivaceum* | Wood Saprotroph | | | 0.00 | | 0.25 | 0.32 | 65.32 |
| 27826935802a898d6e376af873dfac8d | *Cladosporium herbarum* | Animal Pathogen-Endophyte-Lichen Parasite-Plant Pathogen-Wood Saprotroph | | | 0.00 | | 0.25 | 0.31 | 65.63 |
| 4a579adf11a240fae038dcb6e2b0c8a1 | Hyphodiscus sp | Plant Saprotroph-Wood Saprotroph | | | 0.00 | | 0.24 | 0.30 | 65.93 |
| 39e5b1410f470c2e242d0c15fc52b0a6 | Unclassified | Animal Pathogen-Plant Pathogen-Undefined Saprotroph | | | 0.00 | | 0.24 | 0.30 | 66.23 |
| 02b2c6a22639ebc34d3725c04190b796 | Unclassified | Unknown | | | 0.00 | | 0.23 | 0.29 | 66.52 |
| 8961ad5df9c942722bca7e937d91734f | *Retiarius bovicornutus* | Wood Saprotroph | | | 0.00 | | 0.23 | 0.29 | 66.81 |
| b48e309dbacca660bc0d56e3b2106ede | Phaeothecaceae sp | Unknown | | | 0.00 | | 0.22 | 0.28 | 67.09 |
| a59a4dbcc53f1b196ae62b6a5f571208 | Filobasidiales sp | Unknown | | | | 0.00 | 0.22 | 0.27 | 67.36 |
| b725769e40f771098ace29ca25e460ad | Phaeococcomyces sp | Undefined Saprotroph | | | | 0.00 | 0.22 | 0.27 | 67.63 |
| 0405a3f31001aecb4f874f84e64a94b3 | *Lophodermium resinosum* | Endophyte-Leaf Saprotroph-Plant Pathogen | | | | 0.00 | 0.21 | 0.27 | 67.90 |
| bccbe04ab93ba82a26a5461eb1a99fc9 | *Endoconidioma populi* | Endophyte-Plant Pathogen-Undefined Saprotroph | | | | 0.00 | 0.21 | 0.27 | 68.17 |
| 5ceab57904d011cc1e38f09659a88c04 | Lophodermium sp | Endophyte-Leaf Saprotroph-Plant Pathogen | | | | 0.00 | 0.21 | 0.26 | 68.43 |
| 30ae6ecc2a11dcfba284bdd6d1152726 | *Lophodermium baculiferum* | Endophyte-Leaf Saprotroph-Plant Pathogen | | | | 0.00 | 0.21 | 0.26 | 68.69 |
| 710943a3854773a1ba7672c8b42e0c2e | *Neophaeomoniella ossiformis* | Plant Pathogen | | | | 0.00 | 0.20 | 0.25 | 68.95 |
| 476bb38fbbdebbf8a02c2d3c01bd1598 | Dothideales sp | Unknown | | | | 0.00 | 0.20 | 0.25 | 69.20 |
| 7c8cbe1a16c69ab994cf26a30384000f | *Taphrina carpini* | Plant Pathogen | | | | 0.00 | 0.20 | 0.25 | 69.45 |
| a73423bf0e6e0fd72a1c00c762aaa44a | *Lophodermium resinosum* | Endophyte-Leaf Saprotroph-Plant Pathogen | | | | 0.00 | 0.19 | 0.24 | 69.69 |
| afb87befb519e2b23bfa1625089b8181 | Unclassified | Endophyte-Leaf Saprotroph-Plant Pathogen | | | | 0.00 | 0.19 | 0.24 | 69.92 |
| d144c7326a804f7f2c413f66829f0e47 | *Phaeococcomyces kinklidomatophilus* | Undefined Saprotroph | | | | 0.00 | 0.19 | 0.23 | 70.16 |
|  |  |  | | | |  |  |  |  |
| ***P. contorta subsp.***  ***latifolia, Sweden (72.73%)*** | |  | | | |  |  |  |  |
| 58dc2388bdf13a3259088459c94d1719 | *Hormonema macrosporum* | Undefined Saprotroph | | | | 0.17 | 7.94 | 10.92 | 10.92 |
| b8a1abd6b37122017f3a26e995958827 | Unclassified | Unknown | | | | 0.05 | 2.89 | 3.97 | 14.89 |
| 486261a00a597d96ea64717266937962 | Unclassified | Unknown | | | | 0.03 | 1.69 | 2.32 | 17.21 |
| 289f6cd604d766d3681f35c0c9b2223f | *Phaeotheca fissurella* | Plant Pathogen-Plant Saprotroph | | | | 0.03 | 1.48 | 2.04 | 19.25 |
| 7d5c3543ac7e5381e048aa732c187e28 | Unclassified | Unknown | | | | 0.02 | 1.45 | 2.00 | 21.25 |
| d23c794d2e3517c216ec1025795aef4a | *Perusta inaequalis* | Undefined Saprotroph | | | | 0.03 | 1.40 | 1.92 | 23.17 |
| 77a3857ab7c279cf4dcfbd0a9cc4880c | *Phaeococcomyces mexicanus* | Undefined Saprotroph | | | | 0.03 | 1.20 | 1.65 | 24.83 |
| 5a82b07762b024b509ef77c610c80972 | Unclassified | Unknown | | | | 0.02 | 1.01 | 1.38 | 26.21 |
| ee9d0983eed820a458b88ec0a0e900ae | *Phaeotheca fissurella* | Plant Pathogen-Plant Saprotroph | | | | 0.01 | 0.97 | 1.33 | 27.54 |
| ab11097130860a0d0299a31abb394fa2 | *Neophaeomoniella constricta* | Plant Pathogen | | | | 0.01 | 0.94 | 1.29 | 28.83 |
| f82bbdca7a15782b6e9d9ebc4033d01f | Unclassified | Unknown | | | | 0.01 | 0.90 | 1.24 | 30.07 |
| c80c9a2f4659bdca509acb8ccdfc3144 | Unclassified | Unknown | | | | 0.01 | 0.90 | 1.23 | 31.30 |
| 9c9b7bc54bf6cd74f9547eaf2fa4b39c | *Lapidomyces hispanicus* | Animal Pathogen-Plant Pathogen-Undefined Saprotroph | | | | 0.01 | 0.86 | 1.18 | 32.49 |
| 4a579adf11a240fae038dcb6e2b0c8a1 | Hyphodiscus sp | Plant Saprotroph-Wood Saprotroph | | | | 0.01 | 0.81 | 1.11 | 33.60 |
| 5800fb89efe45f720676a47ebf6ed42c | Unclassified | Unknown | | | | 0.01 | 0.73 | 1.00 | 34.60 |
| b426507b1db4c7239f3d47cb101f3991 | Unclassified | Unknown | | | | 0.01 | 0.72 | 0.98 | 35.59 |
| 082de607370fde45e9a1df5a7924e967 | Unclassified | Undefined Saprotroph | | | | 0.02 | 0.71 | 0.98 | 36.56 |
| 67849dd89e37a608a414869c5eb5e0c1 | Phaeotheca sp | Plant Pathogen-Plant Saprotroph | | | | 0.01 | 0.66 | 0.91 | 37.47 |
| f8bc9f50a5f29dea3ae1fe4b0116b3e8 | Hyphodiscus sp | Plant Saprotroph-Wood Saprotroph | | | | 0.01 | 0.66 | 0.91 | 38.38 |
| 965922e455352dbbf8003bfa94ea61b0 | Lichenostigmatales sp | Unknown | | | | 0.01 | 0.65 | 0.89 | 39.27 |
| f1705d5fe0672e785bb360b40c5d723e | Phaffia sp | Undefined Saprotroph | | | | 0.01 | 0.63 | 0.87 | 40.14 |
| 7f08b5ae6a138693a342d18fe1a4afaf | Unclassified | Unknown | | | | 0.01 | 0.61 | 0.83 | 40.97 |
| 24c60d845258ca3c3cdddd735e11449b | Epithamnolia sp | Lichen Parasite | | | | 0.01 | 0.57 | 0.78 | 41.75 |
| 4420a62534309780db450bf0596e06cb | *Lophodermium conigenum* | Endophyte-Leaf Saprotroph-Plant Pathogen | | | | 0.01 | 0.55 | 0.75 | 42.50 |
| 429953e13effc806776228d7fef9f13c | Lapidomyces sp | Animal Pathogen-Plant Pathogen-Undefined Saprotroph | | | | 0.01 | 0.52 | 0.72 | 43.22 |
| b8b707b5f861bb6e1a3ba2b9cfbf7fe3 | Fungi sp | Unknown | | | | 0.01 | 0.52 | 0.71 | 43.93 |
| 2bd4fd75488802a5d13a4d8776a171ae | *Amphosoma atroolivaceum* | Wood Saprotroph | | | | 0.01 | 0.52 | 0.71 | 44.64 |
| 59ed2297db27ba01b7241ae9173cefc3 | Unclassified | Unknown | | | | 0.01 | 0.47 | 0.64 | 45.29 |
| bc5824c1cd8a520a6f3ec74e14d51ebf | Mrakiaceae sp | Unknown | | | | 0.00 | 0.46 | 0.63 | 45.92 |
| a24a6611c7a6762ac5f5edcddbfff58f | *Phaeotheca fissurella* | Plant Pathogen-Plant Saprotroph | | | | 0.01 | 0.46 | 0.63 | 46.55 |
| cdcdcac8b6ce933d156e42e5d5e9d266 | Teloschistaceae sp | Lichenized | | | | 0.01 | 0.44 | 0.61 | 47.16 |
| 710943a3854773a1ba7672c8b42e0c2e | *Neophaeomoniella ossiformis* | Plant Pathogen | | | | 0.01 | 0.43 | 0.60 | 47.76 |
| 12a4c9f2c63f0bf7d366431144d21278 | Unclassified | Unknown | | | | 0.01 | 0.43 | 0.59 | 48.35 |
| 35f1cedf646ad1ff5d15caf5fe097494 | *Phaeotheca fissurella* | Plant Pathogen-Plant Saprotroph | | | | 0.01 | 0.43 | 0.59 | 48.93 |
| 2182387b6fe3b5b71fd24f42d57c7192 | Dothideales sp | Unknown | | | | 0.01 | 0.41 | 0.57 | 49.50 |
| 1158fbb5e610e5c00f960342f30efe4f | Lichenostigmatales sp | Unknown | | | | 0.01 | 0.41 | 0.57 | 50.07 |
| 58374769a8ac08b65e17d44997a5b4cf | *Aequabiliella palatina* | Plant Pathogen | | | | 0.00 | 0.41 | 0.56 | 50.63 |
| 4ab2efb71bb4f523c0bbcec242c92557 | Rhytismatales sp | Unknown | | | | 0.00 | 0.40 | 0.55 | 51.19 |
| 1a3953160684ec935b6beff95c4ef4b9 | Hyphodiscus sp | Plant Saprotroph-Wood Saprotroph | | | | 0.00 | 0.39 | 0.54 | 51.73 |
| 1bb573fd3435b3a745f025793be8f290 | Unclassified | Epiphyte-Plant Pathogen | | | | 0.01 | 0.39 | 0.54 | 52.27 |
| 9bc4b43cd9ac0efd9c1163bbff1e5208 | Calycina sp | Plant Saprotroph-Wood Saprotroph | | | | 0.00 | 0.38 | 0.53 | 52.80 |
| 2bae77c3c87daa6d002730d61da23d34 | *Allantophomopsiella pseudotsugae* | Plant Pathogen | | | | 0.00 | 0.38 | 0.53 | 53.32 |
| b3fc0f62ae7fe0ee6e1d884ebe0fbdb3 | Phaeothecaceae sp | Unknown | | | | 0.01 | 0.38 | 0.52 | 53.84 |
| 25da20be18ddbf9fdb4b9dc7a94737d1 | Mrakiaceae sp | Unknown | | | | 0.00 | 0.37 | 0.51 | 54.35 |
| 1339ced75e437b40601ea96cacdddf42 | *Neophaeomoniella constricta* | Plant Pathogen | | | | 0.00 | 0.36 | 0.49 | 54.84 |
| e08503cf4f976fa7f12fd6be62895312 | *Lapidomyces hispanicus* | Animal Pathogen-Plant Pathogen-Undefined Saprotroph | | | | 0.00 | 0.34 | 0.47 | 55.31 |
| face80ed68bee212ea327c1f15913b62 | Unclassified | Unknown | | | | 0.00 | 0.34 | 0.47 | 55.78 |
| f1feaae21e5046005e5152db40b2f04b | Subulispora sp | Undefined Saprotroph | | | | 0.00 | 0.33 | 0.46 | 56.24 |
| 8f153a54e526c8cbae0b6a7122212221 | Mrakiaceae sp | Unknown | | | | 0.00 | 0.32 | 0.45 | 56.68 |
| c7b970121cecfe8445beeb7d5c2c22ef | Exobasidium sp | Plant Pathogen | | | | 0.00 | 0.32 | 0.44 | 57.13 |
| 4645decdd4869563ef9f92f1c1d0a8bf | *Lophodermium conigenum* | Endophyte-Leaf Saprotroph-Plant Pathogen | | | | 0.00 | 0.32 | 0.44 | 57.57 |
| 2f5087ee4f8a2e21b74401ef4b388d7d | Capnodiales sp | Unknown | | | | 0.01 | 0.32 | 0.43 | 58.00 |
| e546c7e1071baa9d1545ee65b8860386 | *Lophodermium conigenum* | Endophyte-Leaf Saprotroph-Plant Pathogen | | | | 0.00 | 0.32 | 0.43 | 58.44 |
| 7c62f432a2eb120c84a16f59b9184e0a | Hyphodiscus sp | Plant Saprotroph-Wood Saprotroph | | | | 0.00 | 0.31 | 0.43 | 58.87 |
| 88cac7c6573ce2acdc30dbe2424d27d0 | Unclassified | Unknown | | | | 0.00 | 0.30 | 0.41 | 59.28 |
| fa616949df1cfe1d75aaa27f24d52f74 | *Scoliciosporum umbrinum* | Lichen Parasite-Lichenized | | | | 0.00 | 0.29 | 0.40 | 59.68 |
| 5342acf32e334c46156e7519fba52ced | *Epibryon interlamellare* | Plant Pathogen | | | | 0.00 | 0.29 | 0.40 | 60.08 |
| 32885d91eca108fb437de1d0ac1549a2 | Phaffia sp | Undefined Saprotroph | | | | 0.00 | 0.28 | 0.38 | 60.46 |
| 8961ad5df9c942722bca7e937d91734f | *Retiarius bovicornutus* | Wood Saprotroph | | | | 0.00 | 0.27 | 0.37 | 60.82 |
| 5e426fb5dedb7092c4fed497650285aa | Unclassified | Unknown | | | | 0.00 | 0.25 | 0.34 | 61.16 |
| d01897ec573cf475d54dcec694f354b6 | *Phaeococcomyces mexicanus* | Undefined Saprotroph | | | | 0.00 | 0.25 | 0.34 | 61.50 |
| 2634f00bfa947c672e2bb00a89ad5dc1 | Curvibasidium sp | Epiphyte-Plant Saprotroph-Undefined Saprotroph-Wood Saprotroph | | | | 0.00 | 0.24 | 0.33 | 61.83 |
| 27a20bf3cfc67a5c1039c12b4b9cd4a3 | *Lophodermium pinastri* | Endophyte-Leaf Saprotroph-Plant Pathogen | | | | 0.00 | 0.24 | 0.33 | 62.16 |
| abc9e9ea49c43b4429704fbf4241cd1a | Unclassified | Unknown | | | | 0.00 | 0.24 | 0.33 | 62.49 |
| bb627eccd35963729dfd8de593345acd | Unclassified | Unknown | | | | 0.00 | 0.24 | 0.33 | 62.82 |
| 0d96521fc9e17f316bff93e54778c2ce | Unclassified | Animal Pathogen-Plant Pathogen-Undefined Saprotroph | | | | 0.00 | 0.23 | 0.32 | 63.14 |
| 2b16246445c0fad71ba3b633972ea17b | *Lachnellula hyalina* | Plant Saprotroph-Wood Saprotroph | | | | 0.00 | 0.23 | 0.32 | 63.46 |
| 771f98f51f88fb83237fe6ea46176036 | Unclassified | Unknown | | | | 0.00 | 0.23 | 0.31 | 63.77 |
| d0f5c5ce6846a5f18e842202e9a2cf45 | Chaetothyriales sp | Unknown | | | | 0.00 | 0.22 | 0.31 | 64.08 |
| c730b8029b089f51ffacf09171ab4d4c | Unclassified | Unknown | | | | 0.00 | 0.22 | 0.31 | 64.38 |
| 9dd6ba8353cf95fde9560701cff8026e | Cryptococcus sp | Fungal Parasite-Undefined Saprotroph | | | | 0.00 | 0.22 | 0.31 | 64.69 |
| 95598f4668053264920383eebd8f22b7 | Chaetothyriales sp | Unknown | | | | 0.00 | 0.22 | 0.31 | 65.00 |
| 391d81f93f567d2fc064b39d91b9b02d | Unclassified | Animal Pathogen-Plant Pathogen-Undefined Saprotroph | | | | 0.00 | 0.22 | 0.30 | 65.30 |
| a624140ed3cd36997a1de589e9d699ad | Unclassified | Unknown | | | | 0.00 | 0.22 | 0.30 | 65.60 |
| d2db664e03b6ee4fae4c7f99a4af3976 | Unclassified | Unknown | | | | 0.00 | 0.22 | 0.30 | 65.90 |
| 9b27efdfe99dc7d399d1ff8f5aed23e2 | *Hormonema macrosporum* | Undefined Saprotroph | | | | 0.00 | 0.22 | 0.30 | 66.20 |
| fceb64ae87d7550c28beca51a2bc7a07 | Unclassified | Unknown | | | | 0.00 | 0.21 | 0.29 | 66.48 |
| 9b3eceefcb17a2b13e5f2c555ba603a3 | *Lophodermium conigenum* | Endophyte-Leaf Saprotroph-Plant Pathogen | | | | 0.00 | 0.21 | 0.29 | 66.77 |
| 31422f54cd593a08bd6d6fd730d6bf1d | Unclassified | Unknown | | | | 0.00 | 0.20 | 0.28 | 67.05 |
| 7af4f38bba6818ec1410750a33f897fd | Exobasidium sp | Plant Pathogen | | | | 0.00 | 0.20 | 0.28 | 67.32 |
| 953957fa0e77ad26df32bf48837c5693 | Unclassified | Animal Pathogen-Plant Pathogen-Undefined Saprotroph | | | | 0.00 | 0.20 | 0.27 | 67.60 |
| 8a16f1e7d98b1cd4896826b9d59c1c88 | Unclassified | Unknown | | | | 0.00 | 0.20 | 0.27 | 67.87 |
| 5b9c21feabf050582a54f3226c34805b | *Cladosporium basi-inflatum* | Animal Pathogen-Endophyte-Lichen Parasite-Plant Pathogen-Wood Saprotroph | | | | 0.00 | 0.20 | 0.27 | 68.14 |
| b8f10b5957bf04a13a884b1ad7524ec7 | *Phaeococcomyces mexicanus* | Undefined Saprotroph | | | | 0.00 | 0.20 | 0.27 | 68.41 |
| 640acd5715c6cfccde8101247e1cc837 | Unclassified | Unknown | | | | 0.00 | 0.20 | 0.27 | 68.68 |
| 77be01ade270d2acb690596ffa3cf6a7 | *Lophodermium conigenum* | Endophyte-Leaf Saprotroph-Plant Pathogen | | | | 0.00 | 0.19 | 0.27 | 68.95 |
| f0b6b9a5f539cd3febbfa7a82f5cf351 | Unclassified | Unknown | | | | 0.00 | 0.19 | 0.26 | 69.20 |
| 3b59d5a001429c7c694901a837903b64 | Unclassified | Unknown | | | | 0.00 | 0.18 | 0.25 | 69.46 |
| 3f26e13ec6a0f38a2c0562af9fbe961c | *Hormonema macrosporum* | Undefined Saprotroph | | | | 0.00 | 0.18 | 0.25 | 69.71 |
| 8feead68fb63ad67dd119383d26306ec | Chaetothyriales sp | Unknown | | | | 0.00 | 0.18 | 0.25 | 69.96 |
| aa9ae168bf411d73d9cc80a2e7661c23 | Unclassified | Unknown | | | | 0.00 | 0.18 | 0.25 | 70.21 |
|  |  |  | | | |  |  |  |  |
| ***P. contorta subsp. murrayana, USA (79.23%)*** | |  | | | |  |  |  |  |
| fcce34aef79c07cddb8c73bbbb2af57a | *Phaeotheca fissurella* | Plant Pathogen-Plant Saprotroph | | | | 0.12 | 5.88 | 7.43 | 7.43 |
| dc39b76173a46deb54b7d66136ae2dca | *Hormonema macrosporum* | Undefined Saprotroph | | | | 0.09 | 5.64 | 7.11 | 14.54 |
| f93af6ffe0060096694b89af1bb46596 | *Lophodermium baculiferum* | Endophyte-Leaf Saprotroph-Plant Pathogen | | | | 0.04 | 2.58 | 3.26 | 17.80 |
| f363b09c0bb1c3ab378305888d004697 | Rhytismatales sp | Unknown | | | | 0.03 | 1.87 | 2.36 | 20.16 |
| 8cbafb6aca8ca31b83c00f6928e2c79c | *Lophodermium baculiferum* | Endophyte-Leaf Saprotroph-Plant Pathogen | | | | 0.02 | 1.46 | 1.85 | 22.01 |
| 93fcf42d33392baa9b9cc452a220764a | *Lecanora laxa* | Lichen Parasite-Lichenized | | | | 0.02 | 1.23 | 1.55 | 23.56 |
| d0dc7a2aa2aec4c2eb66ac71da86f42e | *Phaeosclera dematioides* | Plant Saprotroph | | | | 0.01 | 1.20 | 1.51 | 25.08 |
| a0524e603c6ef8257818495a9e400421 | Unclassified | Unknown | | | | 0.02 | 1.19 | 1.50 | 26.58 |
| 362d7d5bda6bdffd8764fe90da147f48 | *Meristemomyces arctostaphyli* | Animal Pathogen-Plant Pathogen-Undefined Saprotroph | | | | 0.02 | 1.19 | 1.50 | 28.08 |
| 4ab2efb71bb4f523c0bbcec242c92557 | Rhytismatales sp | Unknown | | | | 0.01 | 1.13 | 1.42 | 29.50 |
| 77a3857ab7c279cf4dcfbd0a9cc4880c | *Phaeococcomyces mexicanus* | Undefined Saprotroph | | | | 0.01 | 1.02 | 1.29 | 30.79 |
| 34d583ca616ed72b44fd14616b1fb217 | Phaeothecaceae sp | Unknown | | | | 0.01 | 0.94 | 1.19 | 31.98 |
| c625ee54c7eeaa128a8d8c2cd8ae8e0f | Unclassified | Animal Pathogen-Plant Pathogen-Undefined Saprotroph | | | | 0.02 | 0.88 | 1.12 | 33.09 |
| aff8b0eb211ef9900fbf9067b156fecf | Unclassified | Endophyte-Leaf Saprotroph-Plant Pathogen | | | | 0.01 | 0.85 | 1.07 | 34.17 |
| 9c79312f9bd2f58f33fd7614a585c5e6 | *Antarctolichenia onofrii* | Unknown | | | | 0.01 | 0.79 | 1.00 | 35.17 |
| 150e291f20f885501cf6731e9deede09 | Sphaeropezia sp | Lichen Parasite | | | | 0.01 | 0.75 | 0.94 | 36.11 |
| 10344dd6d6317f1f5448a7f74c0b8346 | Perusta sp | Undefined Saprotroph | | | | 0.01 | 0.74 | 0.94 | 37.05 |
| d622e5628760d989faa92b205de55fe4 | Myriangiales sp | Unknown | | | | 0.01 | 0.74 | 0.93 | 37.98 |
| 4b2951baa03804efd7a8b08a2daa08a8 | *Antarctolichenia onofrii* | Unknown | | | | 0.01 | 0.72 | 0.90 | 38.89 |
| fe63ea2f753a320ce3f0f2038456367d | Unclassified | Unknown | | | | 0.01 | 0.71 | 0.90 | 39.78 |
| 63d39dae8cc1d0a7887f4acaa42c4e9e | Epithamnolia sp | Lichen Parasite | | | | 0.01 | 0.67 | 0.85 | 40.63 |
| 7cb01bda84001798e08e6fea49e318b6 | Unclassified | Unknown | | | | 0.01 | 0.66 | 0.84 | 41.47 |
| 7ac13fd06d3d4d77234e50e1a6e39baa | Unclassified | Unknown | | | | 0.01 | 0.66 | 0.83 | 42.30 |
| 0d96521fc9e17f316bff93e54778c2ce | Unclassified | Animal Pathogen-Plant Pathogen-Undefined Saprotroph | | | | 0.01 | 0.66 | 0.83 | 43.13 |
| f44779cbd05d60be8fcee9afdaac8170 | Unclassified | Unknown | | | | 0.01 | 0.63 | 0.80 | 43.92 |
| c346b54d60079993e8b1d25d9b232d7e | Unclassified | Unknown | | | | 0.01 | 0.58 | 0.74 | 44.66 |
| bf667d423fe19f33589956bbbccc5846 | Phaeothecaceae sp | Unknown | | | | 0.01 | 0.58 | 0.73 | 45.39 |
| 0de22bd79885234c642fc04a6597241f | Sphaeropezia sp | Lichen Parasite | | | | 0.01 | 0.57 | 0.72 | 46.11 |
| 4a10532f2ee08417a0719aecc6e1f4fc | Rhytismatales sp | Unknown | | | | 0.01 | 0.54 | 0.69 | 46.80 |
| 358926fa1f5b50114870629f59748743 | *Neophaeomoniella constricta* | Plant Pathogen | | | | 0.01 | 0.54 | 0.68 | 47.48 |
| e4406cfb2c586b13cbfd716c4ada2d76 | *Lecanora laxa* | Lichen Parasite-Lichenized | | | | 0.01 | 0.53 | 0.67 | 48.15 |
| 289f6cd604d766d3681f35c0c9b2223f | *Phaeotheca fissurella* | Plant Pathogen-Plant Saprotroph | | | | 0.01 | 0.53 | 0.67 | 48.82 |
| 5eac6fbad962e720590518a62e7c5cb0 | Unclassified | Endophyte-Leaf Saprotroph-Plant Pathogen | | | | 0.01 | 0.50 | 0.63 | 49.46 |
| eef0a7478166d3890602381cf406d374 | Epithamnolia sp | Lichen Parasite | | | | 0.01 | 0.49 | 0.62 | 50.07 |
| 7e7e3651ca5b16042b98e03c56eeb79b | *Neophaeomoniella constricta* | Plant Pathogen | | | | 0.01 | 0.48 | 0.60 | 50.67 |
| ee972ea7a5ec75f8562e6e4344c6a957 | Unclassified | Animal Pathogen-Plant Pathogen-Undefined Saprotroph | | | | 0.01 | 0.47 | 0.60 | 51.27 |
| a116efea99d37e577007dc78589621e9 | *Meristemomyces frigidus* | Animal Pathogen-Plant Pathogen-Undefined Saprotroph | | | | 0.00 | 0.46 | 0.59 | 51.86 |
| abd6ee13838dccd51e8b0c9c480a52e7 | Unclassified | Endophyte-Leaf Saprotroph-Plant Pathogen | | | | 0.01 | 0.46 | 0.58 | 52.44 |
| 5b298cafacf5cd9cc90ff08c2102773f | Sphaeropezia sp | Lichen Parasite | | | | 0.00 | 0.45 | 0.57 | 53.00 |
| 46436ba617f9afcc1af3b28d01412195 | *Neophaeomoniella constricta* | Plant Pathogen | | | | 0.00 | 0.45 | 0.57 | 53.57 |
| 8d56551c76959c22539488da29124ff4 | Unclassified | Endophyte-Leaf Saprotroph-Plant Pathogen | | | | 0.00 | 0.45 | 0.56 | 54.14 |
| 183531e08976c39e066a03dfe789042e | Unclassified | Unknown | | | | 0.00 | 0.45 | 0.56 | 54.70 |
| 8a0816195a4a3b26c0a7f71fa6c38a67 | Pseudotaeniolina sp | Animal Pathogen-Plant Pathogen-Undefined Saprotroph | | | | 0.01 | 0.43 | 0.55 | 55.25 |
| 142925e4aea7cfecc2d457dc28e9481f | *Lophodermium baculiferum* | Endophyte-Leaf Saprotroph-Plant Pathogen | | | | 0.00 | 0.41 | 0.52 | 55.77 |
| 7d165e68089348b3240935f4da8897e8 | *Hormonema macrosporum* | Undefined Saprotroph | | | | 0.00 | 0.39 | 0.50 | 56.27 |
| 24cd5af0977e68168fd4dcadcc086dc1 | Pseudotaeniolina sp | Animal Pathogen-Plant Pathogen-Undefined Saprotroph | | | | 0.01 | 0.39 | 0.49 | 56.75 |
| 5f75a72fe4b253759f70785847b22018 | Lecanora sp | Lichen Parasite-Lichenized | | | | 0.00 | 0.39 | 0.49 | 57.24 |
| e08503cf4f976fa7f12fd6be62895312 | *Lapidomyces hispanicus* | Animal Pathogen-Plant Pathogen-Undefined Saprotroph | | | | 0.00 | 0.38 | 0.48 | 57.72 |
| 2ac7cc94d8ff5b0ecaabf041d950245e | Sphaeropezia sp | Lichen Parasite | | | | 0.00 | 0.38 | 0.48 | 58.20 |
| 042fd39dd971f35c63b646ee9d60838a | Unclassified | Unknown | | | | 0.00 | 0.36 | 0.45 | 58.65 |
| f0ca0fdfdabc30530a994821655aaa11 | *Antarctolichenia onofrii* | Unknown | | | | 0.00 | 0.35 | 0.45 | 59.10 |
| 60e0962da6fc4c2f94bb1c6741fae560 | Sphaeropezia sp | Lichen Parasite | | | | 0.00 | 0.35 | 0.45 | 59.54 |
| c12772e76b3b39f5ca3f262a2dc7ac5f | Unclassified | Unknown | | | | 0.00 | 0.35 | 0.44 | 59.98 |
| 53d78514decb018bdf4107b9720c5c04 | Baeomycetales sp | Unknown | | | | 0.00 | 0.34 | 0.43 | 60.42 |
| 513e95005020daf3fbcb803c048d3b2f | *Phaeosclera dematioides* | Plant Saprotroph | | | | 0.00 | 0.34 | 0.43 | 60.85 |
| 3b26447143390850a37ce3facf0ac30e | Phaeothecaceae sp | Unknown | | | | 0.00 | 0.34 | 0.43 | 61.28 |
| bb627eccd35963729dfd8de593345acd | Unclassified | Unknown | | | | 0.00 | 0.34 | 0.43 | 61.71 |
| c912c671ae548255e3bc666a1c337dbc | *Phaeotheca fissurella* | Plant Pathogen-Plant Saprotroph | | | | 0.00 | 0.33 | 0.41 | 62.12 |
| 3486b94cc33777f26921e2c030c55ea2 | Unclassified | Unknown | | | | 0.00 | 0.31 | 0.40 | 62.52 |
| f43e493de90779c66916cc9d7de0eca5 | Unclassified | Animal Pathogen-Plant Pathogen-Undefined Saprotroph | | | | 0.00 | 0.31 | 0.39 | 62.91 |
| 27826935802a898d6e376af873dfac8d | *Cladosporium herbarum* | Animal Pathogen-Endophyte-Lichen Parasite-Plant Pathogen-Wood Saprotroph | | | | 0.00 | 0.30 | 0.38 | 63.29 |
| a9e61c18c3c25cb23f92788d4fef7afe | Unclassified | Unknown | | | | 0.00 | 0.29 | 0.36 | 63.65 |
| bfcf8e6e91d4e90a0b3afb1c0b6f0fda | Unclassified | Unknown | | | | 0.00 | 0.29 | 0.36 | 64.01 |
| 0854a87d12d9c42e610cf510dbb2ee24 | *Phaeotheca fissurella* | Plant Pathogen-Plant Saprotroph | | | | 0.00 | 0.28 | 0.36 | 64.37 |
| 36a26ff80fa6c6fa769e1227d7003d0c | Phaeothecaceae sp | Unknown | | | | 0.00 | 0.28 | 0.36 | 64.73 |
| 03a43e4f5151ea1b666fb36763cac809 | Unclassified | Unknown | | | | 0.00 | 0.28 | 0.36 | 65.09 |
| 1d0f6f6b914803aab4be77e1c7c76851 | *Antarctolichenia onofrii* | Unknown | | | | 0.00 | 0.28 | 0.35 | 65.44 |
| b93a0b1e8dd2a7636775f2495a7fa107 | Unclassified | Endophyte-Leaf Saprotroph-Plant Pathogen | | | | 0.00 | 0.28 | 0.35 | 65.80 |
| 3bab2ac801202ff79dfdcc21697ced33 | Unclassified | Unknown | | | | 0.00 | 0.28 | 0.35 | 66.15 |
| 6d7941d8bedd11ccf1a9f68807851512 | Lecanora sp | Lichen Parasite-Lichenized | | | | 0.00 | 0.27 | 0.34 | 66.49 |
| 4aab35f581814aafb6203fea7cbd54e3 | Unclassified | Unknown | | | | 0.00 | 0.26 | 0.33 | 66.82 |
| 7a6e23fa2ebf26aa50cf1618e2f48287 | *Hormonema macrosporum* | Undefined Saprotroph | | | | 0.00 | 0.26 | 0.32 | 67.15 |
| ad67f8db00f1f4488acf21de089d8756 | Unclassified | Unknown | | | | 0.00 | 0.25 | 0.32 | 67.47 |
| 7872bcfab94d7b5d097b2b2f19d2a7a0 | Unclassified | Endophyte-Leaf Saprotroph-Plant Pathogen | | | | 0.00 | 0.25 | 0.32 | 67.79 |
| 107fd7342806e11de908c1586d655e93 | *Neophaeomoniella constricta* | Plant Pathogen | | | | 0.00 | 0.25 | 0.31 | 68.10 |
| 69f49cc2aa7eadd285ca324137f8ff75 | Unclassified | Lichen Parasite-Lichenized | | | | 0.00 | 0.25 | 0.31 | 68.41 |
| ccdaf9953d735641f5ef8e9351223de2 | Sphaeropezia sp | Lichen Parasite | | | | 0.00 | 0.24 | 0.31 | 68.72 |
| 1da4fdf5f06a9d432db9704cb807eb14 | Sphaeropezia sp | Lichen Parasite | | | | 0.00 | 0.24 | 0.31 | 69.02 |
| d144c7326a804f7f2c413f66829f0e47 | *Phaeococcomyces kinklidomatophilus* | Undefined Saprotroph | | | | 0.00 | 0.22 | 0.28 | 69.30 |
| 987e631965c67fd7b4c0cad0f90d8816 | Dothideales sp | Unknown | | | | 0.00 | 0.21 | 0.26 | 69.56 |
| 092fda4631aa65a4f671503071ffb508 | Phaeothecaceae sp | Unknown | | | | 0.00 | 0.20 | 0.26 | 69.82 |
| 5afe5c69ed40a75fd5d7ed1891b183be | Epithamnolia sp | Lichen Parasite | | | | 0.00 | 0.20 | 0.25 | 70.07 |
|  |  |  | | | |  |  |  |  |
| ***P. contorta subsp. murrayana plantation, Patagonia (70.65%)*** | |  | | | |  |  |  |  |
| fcce34aef79c07cddb8c73bbbb2af57a | *Phaeotheca fissurella* | Plant Pathogen-Plant Saprotroph | | | | 0.10 | 6.26 | 8.86 | 8.86 |
| dc39b76173a46deb54b7d66136ae2dca | *Hormonema macrosporum* | Undefined Saprotroph | | | | 0.10 | 5.59 | 7.91 | 16.77 |
| e2b1d4a0970bd6cb3fbc2797d232de1b | Unclassified | Unknown | | | | 0.06 | 4.54 | 6.42 | 23.19 |
| bcd0b09c8c27da3e19eb508bf3229f79 | Rhytismatales sp | Unknown | | | | 0.08 | 3.91 | 5.54 | 28.72 |
| f363b09c0bb1c3ab378305888d004697 | Rhytismatales sp | Unknown | | | | 0.08 | 3.90 | 5.51 | 34.24 |
| 45b507e2c856652acd919b1511055f2f | Perusta sp | Undefined Saprotroph | | | | 0.05 | 3.76 | 5.32 | 39.55 |
| ee7ce27261257e5115a91c70ba994dec | *Hormonema macrosporum* | Undefined Saprotroph | | | | 0.04 | 3.61 | 5.11 | 44.66 |
| 2a4c0abc2399b8abf3b44b350a9e3a90 | *Lophodermium pinastri* | Endophyte-Leaf Saprotroph-Plant Pathogen | | | | 0.04 | 3.33 | 4.72 | 49.38 |
| 23b1d791a20535d7c0292bf3b90c1cbc | *Exophiala eucalyptorum* | Animal Pathogen-Fungal Parasite-Undefined Saprotroph | | | | 0.04 | 2.69 | 3.81 | 53.19 |
| 5b5fbec61e303fea1d9f02b99fe81b19 | Unclassified | Undefined Saprotroph | | | | 0.04 | 2.58 | 3.65 | 56.84 |
| 51c46e20cc44dfdf6b6846786a9d5a53 | Unclassified | Unknown | | | | 0.03 | 2.22 | 3.14 | 59.98 |
| 77a3857ab7c279cf4dcfbd0a9cc4880c | *Phaeococcomyces mexicanus* | Undefined Saprotroph | | | | 0.04 | 1.83 | 2.59 | 62.57 |
| a62d0809ab987eb5141777a524c55dc2 | Unclassified | Unknown | | | | 0.02 | 1.23 | 1.74 | 64.31 |
| 5ed0874c57a67622ecc07d1e327aaa32 | *Phaeococcomyces mexicanus* | Undefined Saprotroph | | | | 0.01 | 1.00 | 1.42 | 65.72 |
| 4ab2efb71bb4f523c0bbcec242c92557 | Rhytismatales sp | Unknown | | | | 0.01 | 0.93 | 1.31 | 67.03 |
| 4f9a885d21dda65229c8bfe433dd9ffe | *Sarcinomyces crustaceus* | Endophyte | | | | 0.01 | 0.74 | 1.05 | 68.08 |
| 91d45e9c1a1e4f7d6d0ef17bd60b7152 | *Lophodermium pinastri* | Endophyte-Leaf Saprotroph-Plant Pathogen | | | | 0.01 | 0.71 | 1.00 | 69.08 |
| 798e31de55f6d540fbcc1cb79883cab8 | *Ramoconidiophora euphorbiae* | Unknown | | | | 0.01 | 0.70 | 1.00 | 70.08 |
|  |  |  | | | |  |  |  |  |
| ***P. controta subsp. murrayana invasion fronts, Patagonia (75.53%)*** | |  | | | |  |  |  |  |
| fcce34aef79c07cddb8c73bbbb2af57a | *Phaeotheca fissurella* | Plant Pathogen-Plant Saprotroph | | | | 0.19 | 10.91 | 14.45 | 14.45 |
| 45b507e2c856652acd919b1511055f2f | Perusta sp | Undefined Saprotroph | | | | 0.10 | 7.22 | 9.55 | 24.00 |
| dc39b76173a46deb54b7d66136ae2dca | *Hormonema macrosporum* | Undefined Saprotroph | | | | 0.06 | 4.29 | 5.68 | 29.69 |
| 77a3857ab7c279cf4dcfbd0a9cc4880c | *Phaeococcomyces mexicanus* | Undefined Saprotroph | | | | 0.07 | 3.64 | 4.82 | 34.51 |
| 2a4c0abc2399b8abf3b44b350a9e3a90 | *Lophodermium pinastri* | Endophyte-Leaf Saprotroph-Plant Pathogen | | | | 0.04 | 3.37 | 4.46 | 38.97 |
| a62d0809ab987eb5141777a524c55dc2 | Unclassified | Unknown | | | | 0.06 | 3.29 | 4.35 | 43.32 |
| bcd0b09c8c27da3e19eb508bf3229f79 | Rhytismatales sp | Unknown | | | | 0.03 | 2.73 | 3.61 | 46.93 |
| 5ed0874c57a67622ecc07d1e327aaa32 | *Phaeococcomyces mexicanus* | Undefined Saprotroph | | | | 0.03 | 2.43 | 3.21 | 50.14 |
| ee7ce27261257e5115a91c70ba994dec | *Hormonema macrosporum* | Undefined Saprotroph | | | | 0.02 | 2.17 | 2.87 | 53.01 |
| f363b09c0bb1c3ab378305888d004697 | Rhytismatales sp | Unknown | | | | 0.03 | 2.13 | 2.83 | 55.84 |
| 51c46e20cc44dfdf6b6846786a9d5a53 | Unclassified | Unknown | | | | 0.03 | 1.74 | 2.30 | 58.14 |
| 5b5fbec61e303fea1d9f02b99fe81b19 | Unclassified | Undefined Saprotroph | | | | 0.03 | 1.63 | 2.16 | 60.30 |
| e2b1d4a0970bd6cb3fbc2797d232de1b | Unclassified | Unknown | | | | 0.02 | 1.48 | 1.96 | 62.26 |
| d9f8b0e71cf72606088e081b1eb3a407 | *Lophodermium pinastri* | Endophyte-Leaf Saprotroph-Plant Pathogen | | | | 0.01 | 1.16 | 1.53 | 63.79 |
| ee9d0983eed820a458b88ec0a0e900ae | *Phaeotheca fissurella* | Plant Pathogen-Plant Saprotroph | | | | 0.01 | 1.12 | 1.48 | 65.27 |
| 107fd7342806e11de908c1586d655e93 | *Neophaeomoniella constricta* | Plant Pathogen | | | | 0.01 | 0.82 | 1.08 | 66.35 |
| 27826935802a898d6e376af873dfac8d | *Cladosporium herbarum* | Animal Pathogen-Endophyte-Lichen Parasite-Plant Pathogen-Wood Saprotroph | | | | 0.01 | 0.79 | 1.04 | 67.39 |
| 1761b6a14f266c8009c6f25fbe38b01e | *Lophodermium pinastri* | Endophyte-Leaf Saprotroph-Plant Pathogen | | | | 0.01 | 0.71 | 0.94 | 68.34 |
| 6ddb1e67787c9742dec77a50babebeae | *Lichenostigmatales sp* | Unknown | | | | 0.01 | 0.62 | 0.82 | 69.16 |
| bfcf8e6e91d4e90a0b3afb1c0b6f0fda | Unclassified | Unknown | | | | 0.01 | 0.59 | 0.78 | 69.94 |
| df5850014cd9dcfc108851b972a8cc77 | Unclassified | Unknown | | | | 0.01 | 0.56 | 0.74 | 70.68 |

**Table S3**: A list of fungal amplicon sequence variants (ASVs) and their functional classification accounting for the dissimilarity between fungal communities associated with *P. contorta* subsp. *latifolia* at introduced sites in Sweden and paired native range sites in Canada. The total dissimilarity between the tree types was 93.26%, with listed species accounting for 70% of this dissimilarity.

| ASV | Species | Functional classification | Canada average abundance | Sweden average abundance | Average dissimilarity (%) | Dissimilarity contribution (%) | Cumulative dissimilarity contribution (%) |
| --- | --- | --- | --- | --- | --- | --- | --- |
| 58dc2388bdf13a3259088459c94d1719 | *Hormonema macrosporum* | Undefined Saprotroph | 0.00 | 0.17 | 8.30 | 8.90 | 8.90 |
| 31dece8c6d61e8794e2dd50737d339d3 | *Hormonema macrosporum* | Undefined Saprotroph | 0.09 | 0.00 | 4.34 | 4.65 | 13.55 |
| 77a3857ab7c279cf4dcfbd0a9cc4880c | *Phaeococcomyces mexicanus* | Undefined Saprotroph | 0.07 | 0.03 | 2.93 | 3.14 | 16.69 |
| b8a1abd6b37122017f3a26e995958827 | Unclassified | Unknown | 0.00 | 0.05 | 2.35 | 2.51 | 19.21 |
| 1d0e5bdf9d3bde620f87966848cd97d5 | *Lophodermium baculiferum* | Endophyte-Leaf Saprotroph-Plant Pathogen | 0.04 | 0.00 | 2.25 | 2.41 | 21.61 |
| 289f6cd604d766d3681f35c0c9b2223f | *Phaeotheca fissurella* | Plant Pathogen-Plant Saprotroph | 0.04 | 0.03 | 2.06 | 2.20 | 23.82 |
| ed6643c22af17ce8a5a199b3938a3d54 | *Lophodermella concolor* | Endophyte-Leaf Saprotroph-Plant Pathogen | 0.03 | 0.00 | 1.60 | 1.71 | 25.53 |
| 486261a00a597d96ea64717266937962 | Unclassified | Unknown | 0.00 | 0.03 | 1.50 | 1.61 | 27.14 |
| 8cbafb6aca8ca31b83c00f6928e2c79c | *Lophodermium baculiferum* | Endophyte-Leaf Saprotroph-Plant Pathogen | 0.03 | 0.00 | 1.36 | 1.46 | 28.60 |
| c65f504e999765f2ce42ad83da5de55e | *Lophodermium baculiferum* | Endophyte-Leaf Saprotroph-Plant Pathogen | 0.03 | 0.00 | 1.30 | 1.40 | 30.00 |
| d23c794d2e3517c216ec1025795aef4a | *Perusta inaequalis* | Undefined Saprotroph | 0.02 | 0.03 | 1.28 | 1.38 | 31.37 |
| e08503cf4f976fa7f12fd6be62895312 | *Lapidomyces hispanicus* | Animal Pathogen-Plant Pathogen-Undefined Saprotroph | 0.02 | 0.00 | 1.11 | 1.19 | 32.57 |
| 06fdd6a4c255731826571c830e379bb3 | *Lophodermium baculiferum* | Endophyte-Leaf Saprotroph-Plant Pathogen | 0.02 | 0.00 | 1.00 | 1.07 | 33.63 |
| ae387a169b00b249d56547ee9d9fd350 | Mrakiaceae sp | Unknown | 0.02 | 0.00 | 0.99 | 1.06 | 34.69 |
| 5b9c21feabf050582a54f3226c34805b | *Cladosporium basi-inflatum* | Animal Pathogen-Endophyte-Lichen Parasite-Plant Pathogen-Wood Saprotroph | 0.02 | 0.00 | 0.98 | 1.05 | 35.74 |
| 7d5c3543ac7e5381e048aa732c187e28 | Unclassified | Unknown | 0.00 | 0.02 | 0.96 | 1.03 | 36.77 |
| 35f1cedf646ad1ff5d15caf5fe097494 | *Phaeotheca fissurella* | Plant Pathogen-Plant Saprotroph | 0.02 | 0.01 | 0.91 | 0.98 | 37.75 |
| 5a82b07762b024b509ef77c610c80972 | Unclassified | Unknown | 0.00 | 0.02 | 0.88 | 0.94 | 38.69 |
| ee7ce27261257e5115a91c70ba994dec | *Hormonema macrosporum* | Undefined Saprotroph | 0.02 | 0.00 | 0.86 | 0.92 | 39.61 |
| 9c9b7bc54bf6cd74f9547eaf2fa4b39c | *Lapidomyces hispanicus* | Animal Pathogen-Plant Pathogen-Undefined Saprotroph | 0.01 | 0.01 | 0.79 | 0.85 | 40.46 |
| 082de607370fde45e9a1df5a7924e967 | Unclassified | Undefined Saprotroph | 0.00 | 0.02 | 0.76 | 0.82 | 41.27 |
| ab11097130860a0d0299a31abb394fa2 | *Neophaeomoniella constricta* | Plant Pathogen | 0.00 | 0.01 | 0.74 | 0.80 | 42.07 |
| c80c9a2f4659bdca509acb8ccdfc3144 | Unclassified | Unknown | 0.00 | 0.01 | 0.68 | 0.73 | 42.80 |
| 3903170797690df40698e22775f918f9 | Myriangiales sp | Unknown | 0.01 | 0.00 | 0.65 | 0.70 | 43.50 |
| 67849dd89e37a608a414869c5eb5e0c1 | Phaeotheca sp | Plant Pathogen-Plant Saprotroph | 0.00 | 0.01 | 0.59 | 0.64 | 44.13 |
| 4a579adf11a240fae038dcb6e2b0c8a1 | Hyphodiscus sp | Plant Saprotroph-Wood Saprotroph | 0.00 | 0.01 | 0.58 | 0.62 | 44.75 |
| 12a4c9f2c63f0bf7d366431144d21278 | Unclassified | Unknown | 0.01 | 0.01 | 0.56 | 0.60 | 45.35 |
| ee9d0983eed820a458b88ec0a0e900ae | *Phaeotheca fissurella* | Plant Pathogen-Plant Saprotroph | 0.00 | 0.01 | 0.55 | 0.59 | 45.94 |
| f82bbdca7a15782b6e9d9ebc4033d01f | Unclassified | Unknown | 0.00 | 0.01 | 0.55 | 0.59 | 46.53 |
| 142925e4aea7cfecc2d457dc28e9481f | *Lophodermium baculiferum* | Endophyte-Leaf Saprotroph-Plant Pathogen | 0.01 | 0.00 | 0.54 | 0.58 | 47.11 |
| 7f3aa1241c8212141df9a7400c4101da | Unclassified | Unknown | 0.01 | 0.00 | 0.53 | 0.57 | 47.68 |
| 24c60d845258ca3c3cdddd735e11449b | Epithamnolia sp | Lichen Parasite | 0.00 | 0.01 | 0.53 | 0.57 | 48.25 |
| fcce34aef79c07cddb8c73bbbb2af57a | *Phaeotheca fissurella* | Plant Pathogen-Plant Saprotroph | 0.01 | 0.00 | 0.53 | 0.57 | 48.82 |
| 7f08b5ae6a138693a342d18fe1a4afaf | Unclassified | Unknown | 0.00 | 0.01 | 0.48 | 0.52 | 49.34 |
| 5800fb89efe45f720676a47ebf6ed42c | Unclassified | Unknown | 0.00 | 0.01 | 0.46 | 0.49 | 49.83 |
| 0d96521fc9e17f316bff93e54778c2ce | Unclassified | Animal Pathogen-Plant Pathogen-Undefined Saprotroph | 0.01 | 0.00 | 0.46 | 0.49 | 50.32 |
| 2bae77c3c87daa6d002730d61da23d34 | *Allantophomopsiella pseudotsugae* | Plant Pathogen | 0.00 | 0.00 | 0.45 | 0.48 | 50.80 |
| 9df0cee9028d725c973459e4761c4d72 | Unclassified | Unknown | 0.01 | 0.00 | 0.44 | 0.47 | 51.28 |
| 965922e455352dbbf8003bfa94ea61b0 | Lichenostigmatales sp | Unknown | 0.00 | 0.01 | 0.42 | 0.45 | 51.73 |
| b8b707b5f861bb6e1a3ba2b9cfbf7fe3 | Fungi sp | Unknown | 0.00 | 0.01 | 0.42 | 0.45 | 52.18 |
| f8bc9f50a5f29dea3ae1fe4b0116b3e8 | Hyphodiscus sp | Plant Saprotroph-Wood Saprotroph | 0.00 | 0.01 | 0.42 | 0.45 | 52.63 |
| e4e4a368868f4ba65b9b0f6c18185c83 | Myriangiales sp | Unknown | 0.01 | 0.00 | 0.40 | 0.43 | 53.06 |
| b3fc0f62ae7fe0ee6e1d884ebe0fbdb3 | Phaeothecaceae sp | Unknown | 0.01 | 0.01 | 0.39 | 0.42 | 53.48 |
| f1705d5fe0672e785bb360b40c5d723e | Phaffia sp | Undefined Saprotroph | 0.00 | 0.01 | 0.39 | 0.41 | 53.89 |
| b426507b1db4c7239f3d47cb101f3991 | Unclassified | Unknown | 0.00 | 0.01 | 0.38 | 0.41 | 54.30 |
| 7e7e3651ca5b16042b98e03c56eeb79b | *Neophaeomoniella constricta* | Plant Pathogen | 0.01 | 0.00 | 0.36 | 0.39 | 54.68 |
| cdcdcac8b6ce933d156e42e5d5e9d266 | Teloschistaceae sp | Lichenized | 0.00 | 0.01 | 0.35 | 0.38 | 55.06 |
| 2bd4fd75488802a5d13a4d8776a171ae | *Amphosoma atroolivaceum* | Wood Saprotroph | 0.00 | 0.01 | 0.35 | 0.38 | 55.44 |
| 1bb573fd3435b3a745f025793be8f290 | Unclassified | Epiphyte-Plant Pathogen | 0.00 | 0.01 | 0.35 | 0.37 | 55.81 |
| 26e6e088ab5645b96e3698d5102a4257 | *Lophodermium baculiferum* | Endophyte-Leaf Saprotroph-Plant Pathogen | 0.01 | 0.00 | 0.34 | 0.37 | 56.18 |
| 2182387b6fe3b5b71fd24f42d57c7192 | Dothideales sp | Unknown | 0.00 | 0.01 | 0.33 | 0.36 | 56.54 |
| 710943a3854773a1ba7672c8b42e0c2e | *Neophaeomoniella ossiformis* | Plant Pathogen | 0.00 | 0.01 | 0.33 | 0.35 | 56.89 |
| 931d5d984a4c9edd64e4b9eeef39df5e | Pseudeurotiaceae sp | Plant Saprotroph-Wood Saprotroph | 0.01 | 0.00 | 0.33 | 0.35 | 57.24 |
| a24a6611c7a6762ac5f5edcddbfff58f | *Phaeotheca fissurella* | Plant Pathogen-Plant Saprotroph | 0.00 | 0.01 | 0.33 | 0.35 | 57.59 |
| 891aaffd033d6ab6fc788c6d0800f284 | Unclassified | Plant Pathogen-Undefined Saprotroph | 0.01 | 0.00 | 0.31 | 0.34 | 57.93 |
| 429953e13effc806776228d7fef9f13c | Lapidomyces sp | Animal Pathogen-Plant Pathogen-Undefined Saprotroph | 0.00 | 0.01 | 0.31 | 0.33 | 58.26 |
| 4420a62534309780db450bf0596e06cb | *Lophodermium conigenum* | Endophyte-Leaf Saprotroph-Plant Pathogen | 0.00 | 0.01 | 0.31 | 0.33 | 58.59 |
| 28759794967a7c39dcf158c8409215be | *Hormonema macrosporum* | Undefined Saprotroph | 0.01 | 0.00 | 0.30 | 0.32 | 58.91 |
| d1d39b676469dd14f6e83bbefbc6235a | Unclassified | Unknown | 0.01 | 0.00 | 0.30 | 0.32 | 59.24 |
| b1b64ea48ee82b6f4b3137339dbe8cd5 | *Phaeococcomyces mexicanus* | Undefined Saprotroph | 0.01 | 0.00 | 0.30 | 0.32 | 59.56 |
| ded2771c98ec82002e74d2ab16f661bc | *Lophodermium baculiferum* | Endophyte-Leaf Saprotroph-Plant Pathogen | 0.01 | 0.00 | 0.30 | 0.32 | 59.87 |
| 491231c0fb35030bb280feb5c3a8db2f | Unclassified | Epiphyte | 0.01 | 0.00 | 0.29 | 0.31 | 60.18 |
| 59ed2297db27ba01b7241ae9173cefc3 | Unclassified | Unknown | 0.00 | 0.01 | 0.28 | 0.30 | 60.49 |
| 4fe20330dff299e451c55745c12c7f74 | Capnocheirides sp | Animal Pathogen-Plant Pathogen-Undefined Saprotroph | 0.01 | 0.00 | 0.28 | 0.30 | 60.78 |
| 1158fbb5e610e5c00f960342f30efe4f | Lichenostigmatales sp | Unknown | 0.00 | 0.01 | 0.28 | 0.30 | 61.08 |
| 3307132c681cc78f616436f2ea32743a | *Lophodermium resinosum* | Endophyte-Leaf Saprotroph-Plant Pathogen | 0.01 | 0.00 | 0.28 | 0.30 | 61.38 |
| b251ad15a9ad7f44b68a8ddaa6bcc93d | *Perusta inaequalis* | Undefined Saprotroph | 0.01 | 0.00 | 0.27 | 0.29 | 61.67 |
| b20e3ec8817c42fe8a7fa978962add39 | *Genolevuria tibetensis* | Fungal Parasite-Undefined Saprotroph | 0.01 | 0.00 | 0.26 | 0.28 | 61.94 |
| 58374769a8ac08b65e17d44997a5b4cf | *Aequabiliella palatina* | Plant Pathogen | 0.00 | 0.00 | 0.26 | 0.28 | 62.22 |
| 2f5087ee4f8a2e21b74401ef4b388d7d | Capnodiales sp | Unknown | 0.00 | 0.01 | 0.26 | 0.27 | 62.49 |
| 8961ad5df9c942722bca7e937d91734f | *Retiarius bovicornutus* | Wood Saprotroph | 0.00 | 0.00 | 0.25 | 0.26 | 62.76 |
| 1a3953160684ec935b6beff95c4ef4b9 | Hyphodiscus sp | Plant Saprotroph-Wood Saprotroph | 0.00 | 0.00 | 0.24 | 0.26 | 63.02 |
| bc5824c1cd8a520a6f3ec74e14d51ebf | Mrakiaceae sp | Unknown | 0.00 | 0.00 | 0.24 | 0.26 | 63.27 |
| aff8b0eb211ef9900fbf9067b156fecf | Unclassified | Endophyte-Leaf Saprotroph-Plant Pathogen | 0.00 | 0.00 | 0.24 | 0.26 | 63.53 |
| f93af6ffe0060096694b89af1bb46596 | *Lophodermium baculiferum* | Endophyte-Leaf Saprotroph-Plant Pathogen | 0.00 | 0.00 | 0.24 | 0.25 | 63.78 |
| c7b970121cecfe8445beeb7d5c2c22ef | Exobasidium sp | Plant Pathogen | 0.00 | 0.00 | 0.23 | 0.25 | 64.03 |
| b437379ba9e1476557cc66544d91c5f0 | Unclassified | Endophyte-Plant Pathogen-Undefined Saprotroph | 0.00 | 0.00 | 0.23 | 0.25 | 64.28 |
| 4ab2efb71bb4f523c0bbcec242c92557 | Rhytismatales sp | Unknown | 0.00 | 0.00 | 0.22 | 0.24 | 64.52 |
| 25da20be18ddbf9fdb4b9dc7a94737d1 | Mrakiaceae sp | Unknown | 0.00 | 0.00 | 0.22 | 0.23 | 64.75 |
| a79fd5e4353832dc646678cfbb16ce91 | Unclassified | Unknown | 0.00 | 0.00 | 0.22 | 0.23 | 64.99 |
| 7c6f38afb12b00357089d887d03261d3 | *Lophodermium baculiferum* | Endophyte-Leaf Saprotroph-Plant Pathogen | 0.00 | 0.00 | 0.21 | 0.23 | 65.21 |
| 1339ced75e437b40601ea96cacdddf42 | *Neophaeomoniella constricta* | Plant Pathogen | 0.00 | 0.00 | 0.21 | 0.23 | 65.44 |
| b00637bbb6ec4862e48e22bb19e934e6 | Unclassified | Plant Pathogen | 0.00 | 0.00 | 0.21 | 0.22 | 65.66 |
| face80ed68bee212ea327c1f15913b62 | Unclassified | Unknown | 0.00 | 0.00 | 0.21 | 0.22 | 65.88 |
| 9bc4b43cd9ac0efd9c1163bbff1e5208 | Calycina sp | Plant Saprotroph-Wood Saprotroph | 0.00 | 0.00 | 0.20 | 0.22 | 66.10 |
| 7c62f432a2eb120c84a16f59b9184e0a | Hyphodiscus sp | Plant Saprotroph-Wood Saprotroph | 0.00 | 0.00 | 0.20 | 0.21 | 66.31 |
| 2ba99bc7bf0258de782849a61eaf7a3f | *Lophodermium molitoris* | Endophyte-Leaf Saprotroph-Plant Pathogen | 0.00 | 0.00 | 0.20 | 0.21 | 66.52 |
| eb88a644353f4e6ebd8fd9600bd90e21 | Perusta sp | Undefined Saprotroph | 0.00 | 0.00 | 0.20 | 0.21 | 66.73 |
| 5e426fb5dedb7092c4fed497650285aa | Unclassified | Unknown | 0.00 | 0.00 | 0.19 | 0.21 | 66.94 |
| 54e9cfcbbe8e4cdaf40a61f162c22827 | *Phaeotheca fissurella* | Plant Pathogen-Plant Saprotroph | 0.00 | 0.00 | 0.18 | 0.20 | 67.14 |
| fa616949df1cfe1d75aaa27f24d52f74 | *Scoliciosporum umbrinum* | Lichen Parasite-Lichenized | 0.00 | 0.00 | 0.18 | 0.20 | 67.33 |
| 7e4f4afdcbb411b247a341ca5df104fc | Fungi sp | Unknown | 0.00 | 0.00 | 0.18 | 0.19 | 67.52 |
| 88cac7c6573ce2acdc30dbe2424d27d0 | Unclassified | Unknown | 0.00 | 0.00 | 0.18 | 0.19 | 67.71 |
| 8f153a54e526c8cbae0b6a7122212221 | Mrakiaceae sp | Unknown | 0.00 | 0.00 | 0.18 | 0.19 | 67.90 |
| 5342acf32e334c46156e7519fba52ced | *Epibryon interlamellare* | Plant Pathogen | 0.00 | 0.00 | 0.18 | 0.19 | 68.09 |
| c868ff643495266126e7d26733e7b4bd | *Lophodermium molitoris* | Endophyte-Leaf Saprotroph-Plant Pathogen | 0.00 | 0.00 | 0.18 | 0.19 | 68.28 |
| 744e3313691ebbac20552e3b64401c14 | *Lophodermium resinosum* | Endophyte-Leaf Saprotroph-Plant Pathogen | 0.00 | 0.00 | 0.17 | 0.18 | 68.46 |
| e7de469d4e75395faec40320701a7c39 | *Sarcinomyces crustaceus* | Endophyte | 0.00 | 0.00 | 0.17 | 0.18 | 68.64 |
| f1feaae21e5046005e5152db40b2f04b | Subulispora sp | Undefined Saprotroph | 0.00 | 0.00 | 0.17 | 0.18 | 68.82 |
| 4645decdd4869563ef9f92f1c1d0a8bf | *Lophodermium conigenum* | Endophyte-Leaf Saprotroph-Plant Pathogen | 0.00 | 0.00 | 0.16 | 0.17 | 68.99 |
| 27826935802a898d6e376af873dfac8d | *Cladosporium herbarum* | Animal Pathogen-Endophyte-Lichen Parasite-Plant Pathogen-Wood Saprotroph | 0.00 | 0.00 | 0.16 | 0.17 | 69.16 |
| e546c7e1071baa9d1545ee65b8860386 | *Lophodermium conigenum* | Endophyte-Leaf Saprotroph-Plant Pathogen | 0.00 | 0.00 | 0.16 | 0.17 | 69.33 |
| 04d4624979dff0f162d0cb536c33ed1e | *Lophodermium resinosum* | Endophyte-Leaf Saprotroph-Plant Pathogen | 0.00 | 0.00 | 0.16 | 0.17 | 69.50 |
| 80a9f4f9b3c47f908ad4cb2fe9221239 | *Lophodermella montivaga* | Endophyte-Leaf Saprotroph-Plant Pathogen | 0.00 | 0.00 | 0.16 | 0.17 | 69.67 |
| 8886792f31e4ed4947ea99bcff9aa922 | Unclassified | Unknown | 0.00 | 0.00 | 0.16 | 0.17 | 69.84 |
| a5d23f85a3113a04e48974883d153b77 | *Amphosoma atroolivaceum* | Wood Saprotroph | 0.00 | 0.00 | 0.15 | 0.16 | 70.00 |
| 02b2c6a22639ebc34d3725c04190b796 | Unclassified | Unknown | 0.00 | 0.00 | 0.15 | 0.16 | 70.16 |

**Table S4**: A list of fungal amplicon sequence variants (ASVs) and their functional classification accounting for the dissimilarity between fungal communities associated with *P. contorta* subsp. *murrayana* at introduced sites in Patagonia and paired native range sites in the USA. The total dissimilarity between the tree types was 87.93%, with listed species accounting for 70% of this dissimilarity.

| ASV | Species | Functional classification | USA average abundance | Patagonia average abundance | Average dissimilarity (%) | Dissimilarity contribution (%) | Cumulative dissimilarity contribution (%) |
| --- | --- | --- | --- | --- | --- | --- | --- |
| fcce34aef79c07cddb8c73bbbb2af57a | *Phaeotheca fissurella* | Plant Pathogen-Plant Saprotroph | 0.12 | 0.1 | 6.16 | 7 | 7 |
| dc39b76173a46deb54b7d66136ae2dca | *Hormonema macrosporum* | Undefined Saprotroph | 0.09 | 0.1 | 5.66 | 6.44 | 13.44 |
| bcd0b09c8c27da3e19eb508bf3229f79 | Rhytismatales sp | Unknown | 0 | 0.08 | 4.01 | 4.56 | 18 |
| f363b09c0bb1c3ab378305888d004697 | Rhytismatales sp | Unknown | 0.03 | 0.08 | 3.58 | 4.07 | 22.07 |
| e2b1d4a0970bd6cb3fbc2797d232de1b | Unclassified | Unknown | 0 | 0.06 | 2.83 | 3.21 | 25.28 |
| 45b507e2c856652acd919b1511055f2f | Perusta sp | Undefined Saprotroph | 0 | 0.05 | 2.61 | 2.97 | 28.25 |
| 5b5fbec61e303fea1d9f02b99fe81b19 | Unclassified | Undefined Saprotroph | 0 | 0.04 | 2.18 | 2.48 | 30.73 |
| ee7ce27261257e5115a91c70ba994dec | *Hormonema macrosporum* | Undefined Saprotroph | 0 | 0.04 | 2.1 | 2.39 | 33.12 |
| f93af6ffe0060096694b89af1bb46596 | *Lophodermium baculiferum* | Endophyte-Leaf Saprotroph-Plant Pathogen | 0.04 | 0 | 1.86 | 2.12 | 35.24 |
| 77a3857ab7c279cf4dcfbd0a9cc4880c | *Phaeococcomyces mexicanus* | Undefined Saprotroph | 0.01 | 0.04 | 1.81 | 2.06 | 37.3 |
| 2a4c0abc2399b8abf3b44b350a9e3a90 | *Lophodermium pinastri* | Endophyte-Leaf Saprotroph-Plant Pathogen | 0 | 0.04 | 1.79 | 2.04 | 39.34 |
| 23b1d791a20535d7c0292bf3b90c1cbc | *Exophiala eucalyptorum* | Animal Pathogen-Fungal Parasite-Undefined Saprotroph | 0 | 0.04 | 1.78 | 2.02 | 41.36 |
| 51c46e20cc44dfdf6b6846786a9d5a53 | Unclassified | Unknown | 0 | 0.03 | 1.58 | 1.8 | 43.16 |
| 4ab2efb71bb4f523c0bbcec242c92557 | Rhytismatales sp | Unknown | 0.01 | 0.01 | 1.03 | 1.17 | 44.32 |
| 362d7d5bda6bdffd8764fe90da147f48 | *Meristemomyces arctostaphyli* | Animal Pathogen-Plant Pathogen-Undefined Saprotroph | 0.02 | 0 | 1 | 1.14 | 45.47 |
| 8cbafb6aca8ca31b83c00f6928e2c79c | *Lophodermium baculiferum* | Endophyte-Leaf Saprotroph-Plant Pathogen | 0.02 | 0 | 0.96 | 1.1 | 46.56 |
| a0524e603c6ef8257818495a9e400421 | Unclassified | Unknown | 0.02 | 0 | 0.95 | 1.08 | 47.65 |
| a62d0809ab987eb5141777a524c55dc2 | Unclassified | Unknown | 0 | 0.02 | 0.94 | 1.07 | 48.72 |
| 93fcf42d33392baa9b9cc452a220764a | *Lecanora laxa* | Lichen Parasite-Lichenized | 0.02 | 0 | 0.84 | 0.96 | 49.68 |
| c625ee54c7eeaa128a8d8c2cd8ae8e0f | Unclassified | Animal Pathogen-Plant Pathogen-Undefined Saprotroph | 0.02 | 0 | 0.82 | 0.94 | 50.61 |
| d0dc7a2aa2aec4c2eb66ac71da86f42e | *Phaeosclera dematioides* | Plant Saprotroph | 0.01 | 0 | 0.71 | 0.81 | 51.42 |
| 10344dd6d6317f1f5448a7f74c0b8346 | Perusta sp | Undefined Saprotroph | 0.01 | 0 | 0.64 | 0.73 | 52.15 |
| 9c79312f9bd2f58f33fd7614a585c5e6 | *Antarctolichenia onofrii* | Unknown | 0.01 | 0 | 0.59 | 0.67 | 52.83 |
| aff8b0eb211ef9900fbf9067b156fecf | Unclassified | Endophyte-Leaf Saprotroph-Plant Pathogen | 0.01 | 0 | 0.55 | 0.63 | 53.46 |
| 5ed0874c57a67622ecc07d1e327aaa32 | *Phaeococcomyces mexicanus* | Undefined Saprotroph | 0 | 0.01 | 0.53 | 0.6 | 54.06 |
| 34d583ca616ed72b44fd14616b1fb217 | Phaeothecaceae sp | Unknown | 0.01 | 0 | 0.48 | 0.55 | 54.61 |
| d622e5628760d989faa92b205de55fe4 | Myriangiales sp | Unknown | 0.01 | 0 | 0.47 | 0.53 | 55.15 |
| 107fd7342806e11de908c1586d655e93 | *Neophaeomoniella constricta* | Plant Pathogen | 0 | 0.01 | 0.46 | 0.52 | 55.67 |
| 798e31de55f6d540fbcc1cb79883cab8 | *Ramoconidiophora euphorbiae* | Unknown | 0 | 0.01 | 0.45 | 0.51 | 56.18 |
| 4f9a885d21dda65229c8bfe433dd9ffe | *Sarcinomyces crustaceus* | Endophyte | 0 | 0.01 | 0.45 | 0.51 | 56.69 |
| 4b2951baa03804efd7a8b08a2daa08a8 | *Antarctolichenia onofrii* | Unknown | 0.01 | 0 | 0.44 | 0.51 | 57.2 |
| 7cb01bda84001798e08e6fea49e318b6 | Unclassified | Unknown | 0.01 | 0 | 0.44 | 0.5 | 57.69 |
| ee9d0983eed820a458b88ec0a0e900ae | *Phaeotheca fissurella* | Plant Pathogen-Plant Saprotroph | 0 | 0.01 | 0.43 | 0.49 | 58.18 |
| 150e291f20f885501cf6731e9deede09 | Sphaeropezia sp | Lichen Parasite | 0.01 | 0 | 0.39 | 0.44 | 58.62 |
| b426507b1db4c7239f3d47cb101f3991 | Unclassified | Unknown | 0 | 0.01 | 0.39 | 0.44 | 59.07 |
| bf667d423fe19f33589956bbbccc5846 | Phaeothecaceae sp | Unknown | 0.01 | 0 | 0.38 | 0.44 | 59.5 |
| f44779cbd05d60be8fcee9afdaac8170 | Unclassified | Unknown | 0.01 | 0 | 0.38 | 0.43 | 59.93 |
| 91d45e9c1a1e4f7d6d0ef17bd60b7152 | *Lophodermium pinastri* | Endophyte-Leaf Saprotroph-Plant Pathogen | 0 | 0.01 | 0.37 | 0.42 | 60.35 |
| 0b72f4c2f28dda8c6a3bce4c36fb2f1f | *Neophaeomoniella constricta* | Plant Pathogen | 0 | 0.01 | 0.37 | 0.42 | 60.77 |
| 7ac13fd06d3d4d77234e50e1a6e39baa | Unclassified | Unknown | 0.01 | 0 | 0.37 | 0.42 | 61.18 |
| 0d96521fc9e17f316bff93e54778c2ce | Unclassified | Animal Pathogen-Plant Pathogen-Undefined Saprotroph | 0.01 | 0 | 0.36 | 0.42 | 61.6 |
| 408da00b7419421458c0705e65484289 | *Neophaeomoniella constricta* | Plant Pathogen | 0 | 0.01 | 0.36 | 0.41 | 62.01 |
| 1761b6a14f266c8009c6f25fbe38b01e | *Lophodermium pinastri* | Endophyte-Leaf Saprotroph-Plant Pathogen | 0 | 0.01 | 0.36 | 0.41 | 62.42 |
| fe63ea2f753a320ce3f0f2038456367d | Unclassified | Unknown | 0.01 | 0 | 0.35 | 0.4 | 62.82 |
| bfcf8e6e91d4e90a0b3afb1c0b6f0fda | Unclassified | Unknown | 0 | 0.01 | 0.35 | 0.4 | 63.23 |
| 63d39dae8cc1d0a7887f4acaa42c4e9e | Epithamnolia sp | Lichen Parasite | 0.01 | 0 | 0.35 | 0.4 | 63.63 |
| e4406cfb2c586b13cbfd716c4ada2d76 | *Lecanora laxa* | Lichen Parasite-Lichenized | 0.01 | 0 | 0.34 | 0.39 | 64.02 |
| ee972ea7a5ec75f8562e6e4344c6a957 | Unclassified | Animal Pathogen-Plant Pathogen-Undefined Saprotroph | 0.01 | 0 | 0.34 | 0.39 | 64.41 |
| c346b54d60079993e8b1d25d9b232d7e | Unclassified | Unknown | 0.01 | 0 | 0.32 | 0.37 | 64.78 |
| 349de79bab250ed0b8ce5729769844b4 | Unclassified | Animal Pathogen-Plant Pathogen-Undefined Saprotroph | 0 | 0.01 | 0.32 | 0.36 | 65.14 |
| 4a10532f2ee08417a0719aecc6e1f4fc | Rhytismatales sp | Unknown | 0.01 | 0 | 0.32 | 0.36 | 65.5 |
| 358926fa1f5b50114870629f59748743 | *Neophaeomoniella constricta* | Plant Pathogen | 0.01 | 0 | 0.32 | 0.36 | 65.87 |
| 289f6cd604d766d3681f35c0c9b2223f | *Phaeotheca fissurella* | Plant Pathogen-Plant Saprotroph | 0.01 | 0 | 0.3 | 0.35 | 66.21 |
| 0de22bd79885234c642fc04a6597241f | Sphaeropezia sp | Lichen Parasite | 0.01 | 0 | 0.29 | 0.33 | 66.54 |
| 7e7e3651ca5b16042b98e03c56eeb79b | *Neophaeomoniella constricta* | Plant Pathogen | 0.01 | 0 | 0.28 | 0.32 | 66.86 |
| d9f8b0e71cf72606088e081b1eb3a407 | *Lophodermium pinastri* | Endophyte-Leaf Saprotroph-Plant Pathogen | 0 | 0.01 | 0.28 | 0.31 | 67.17 |
| 1402d01f5676465d58e93d47c1e6841c | Symmetrospora sp | Epiphyte-Plant Saprotroph | 0 | 0.01 | 0.28 | 0.31 | 67.48 |
| 0fe93fff73604319c9a094ee0ec87ee9 | Unclassified | Unknown | 0 | 0.01 | 0.27 | 0.31 | 67.8 |
| 8d6b9dd698949bce34b137398793cd28 | Unclassified | Unknown | 0 | 0.01 | 0.27 | 0.3 | 68.1 |
| 24cd5af0977e68168fd4dcadcc086dc1 | Pseudotaeniolina sp | Animal Pathogen-Plant Pathogen-Undefined Saprotroph | 0.01 | 0 | 0.27 | 0.3 | 68.4 |
| eef0a7478166d3890602381cf406d374 | Epithamnolia sp | Lichen Parasite | 0.01 | 0 | 0.26 | 0.3 | 68.7 |
| abd6ee13838dccd51e8b0c9c480a52e7 | Unclassified | Endophyte-Leaf Saprotroph-Plant Pathogen | 0.01 | 0 | 0.26 | 0.3 | 69 |
| 5eac6fbad962e720590518a62e7c5cb0 | Unclassified | Endophyte-Leaf Saprotroph-Plant Pathogen | 0.01 | 0 | 0.26 | 0.29 | 69.29 |
| 8a0816195a4a3b26c0a7f71fa6c38a67 | Pseudotaeniolina sp | Animal Pathogen-Plant Pathogen-Undefined Saprotroph | 0.01 | 0 | 0.25 | 0.29 | 69.58 |
| 183531e08976c39e066a03dfe789042e | Unclassified | Unknown | 0 | 0 | 0.25 | 0.28 | 69.86 |
| 46436ba617f9afcc1af3b28d01412195 | *Neophaeomoniella constricta* | Plant Pathogen | 0 | 0 | 0.25 | 0.28 | 70.15 |

**Table S5.** The results from PERMANOVA (F) or Welch's t-test (t) or Wilcoxon rank sum (W) test evaluating *Pinus contorta* subsp. *murrayana* needle fungal differences between introduced plantations and invasion fronts in Patagonia. Analysis of community composition was done using PERMANOVA on a Bray-Curtis similarity matrix of fungal taxonomic units identified using PacBio, while richness and relative abundance were analyzed using Welch’s t-tests except for the richness of “others” using the Wilcoxon rank sum test.

|  | F, t, or W | *p* values |
| --- | --- | --- |
| Community composition |  |  |
| All fungi | 10.26 | **0.001** |
| Plant pathogens | 4.78 | **0.002** |
| Endophytes | 3.22 | **0.001** |
| Epiphytes | 1.29 | 0.227 |
| Saprotrophs | 7.57 | **0.001** |
| Others | 1.31 | 0.126 |
| Unknown ecology | 12.92 | **0.001** |
| Richness |  |  |
| All fungi | 0.71 | 0.477 |
| Plant pathogens | 1.65 | 0.100 |
| Endophytes | 2.60 | **0.010** |
| Epiphytes | -0.04 | 0.966 |
| Saprotrophs | 2.49 | **0.014** |
| Others | 309 | 0.210 |
| Unknown ecology | -0.60 | 0.548 |
| Relative abundance |  |  |
| Plant pathogens | 2.37 | **0.019** |
| Endophytes | 0.22 | 0.828 |
| Epiphytes | -2.28 | **0.024** |
| Saprotrophs | 3.07 | **0.003** |
| Others | -2.13 | **0.035** |
| Unknown ecology | -3.57 | **<0.001** |

For community composition data, the F-value is generated using permutation procedures and thus is referred to as a Pseudo-F-value*.* For richness and relative abundance, t-values are used except for the richness of “others” using the W statistic. *p* values in bold are significant at α*=* 0.05.

**Table S6**: A list of fungal amplicon sequence variants (ASVs) and their functional classification accounting for the dissimilarity between fungal communities associated with Patagonia *P. contorta* subsp. *murrayana* trees growing in plantations or invading from the plantations. The total dissimilarity was 78.16%, with listed species accounting for 70% of this dissimilarity.

| ASV | Species | Functional classification | Plantation average abundance | Invasive average abundance | Average dissimilarity (%) | Dissimilarity contribution (%) | Cumulative dissimilarity contribution (%) |
| --- | --- | --- | --- | --- | --- | --- | --- |
| fcce34aef79c07cddb8c73bbbb2af57a | *Phaeotheca fissurella* | Plant Pathogen-Plant Saprotroph | 0.10 | 0.19 | 9.12 | 11.67 | 11.67 |
| 45b507e2c856652acd919b1511055f2f | Perusta sp | Undefined Saprotroph | 0.05 | 0.10 | 5.75 | 7.36 | 19.03 |
| dc39b76173a46deb54b7d66136ae2dca | *Hormonema macrosporum* | Undefined Saprotroph | 0.10 | 0.06 | 5.43 | 6.95 | 25.99 |
| bcd0b09c8c27da3e19eb508bf3229f79 | Rhytismatales sp | Unknown | 0.08 | 0.03 | 4.07 | 5.21 | 31.20 |
| f363b09c0bb1c3ab378305888d004697 | Rhytismatales sp | Unknown | 0.08 | 0.03 | 3.93 | 5.03 | 36.23 |
| 2a4c0abc2399b8abf3b44b350a9e3a90 | *Lophodermium pinastri* | Endophyte-Leaf Saprotroph-Plant Pathogen | 0.04 | 0.04 | 3.33 | 4.26 | 40.49 |
| e2b1d4a0970bd6cb3fbc2797d232de1b | Unclassified | Unknown | 0.06 | 0.02 | 3.30 | 4.22 | 44.71 |
| 77a3857ab7c279cf4dcfbd0a9cc4880c | *Phaeococcomyces mexicanus* | Undefined Saprotroph | 0.04 | 0.07 | 3.09 | 3.95 | 48.66 |
| ee7ce27261257e5115a91c70ba994dec | *Hormonema macrosporum* | Undefined Saprotroph | 0.04 | 0.02 | 2.90 | 3.71 | 52.38 |
| a62d0809ab987eb5141777a524c55dc2 | Unclassified | Unknown | 0.02 | 0.06 | 2.61 | 3.34 | 55.72 |
| 5b5fbec61e303fea1d9f02b99fe81b19 | Unclassified | Undefined Saprotroph | 0.04 | 0.03 | 2.20 | 2.81 | 58.53 |
| 51c46e20cc44dfdf6b6846786a9d5a53 | Unclassified | Unknown | 0.03 | 0.03 | 1.97 | 2.52 | 61.05 |
| 23b1d791a20535d7c0292bf3b90c1cbc | *Exophiala eucalyptorum* | Animal Pathogen-Fungal Parasite-Undefined Saprotroph | 0.04 | 0.00 | 1.79 | 2.29 | 63.34 |
| 5ed0874c57a67622ecc07d1e327aaa32 | *Phaeococcomyces mexicanus* | Undefined Saprotroph | 0.01 | 0.03 | 1.73 | 2.22 | 65.56 |
| ee9d0983eed820a458b88ec0a0e900ae | *Phaeotheca fissurella* | Plant Pathogen-Plant Saprotroph | 0.01 | 0.01 | 0.91 | 1.16 | 66.71 |
| d9f8b0e71cf72606088e081b1eb3a407 | *Lophodermium pinastri* | Endophyte-Leaf Saprotroph-Plant Pathogen | 0.01 | 0.01 | 0.84 | 1.07 | 67.79 |
| 4ab2efb71bb4f523c0bbcec242c92557 | Rhytismatales sp | Unknown | 0.01 | 0.01 | 0.76 | 0.98 | 68.76 |
| 107fd7342806e11de908c1586d655e93 | *Neophaeomoniella constricta* | Plant Pathogen | 0.01 | 0.01 | 0.73 | 0.94 | 69.70 |
| 1761b6a14f266c8009c6f25fbe38b01e | *Lophodermium pinastri* | Endophyte-Leaf Saprotroph-Plant Pathogen | 0.01 | 0.01 | 0.69 | 0.88 | 70.59 |


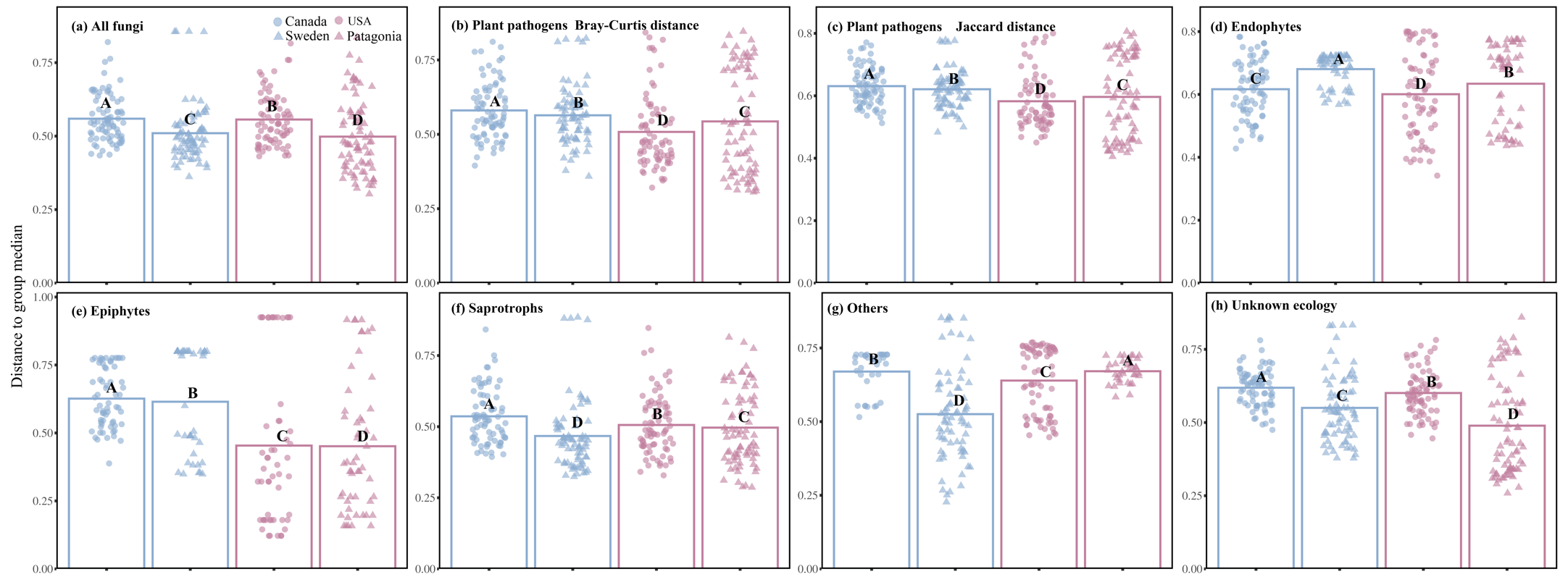


**Figure S1.** Beta-dispersion analysis of fungal communities associated with *Pinus contorta* needles in native (i.e., Canada and the USA) and introduced (i.e., Sweden and Patagonia) plantations. Different letters above bars or across bar segments with the same shade indicate significant differences at α= 0.05.

**
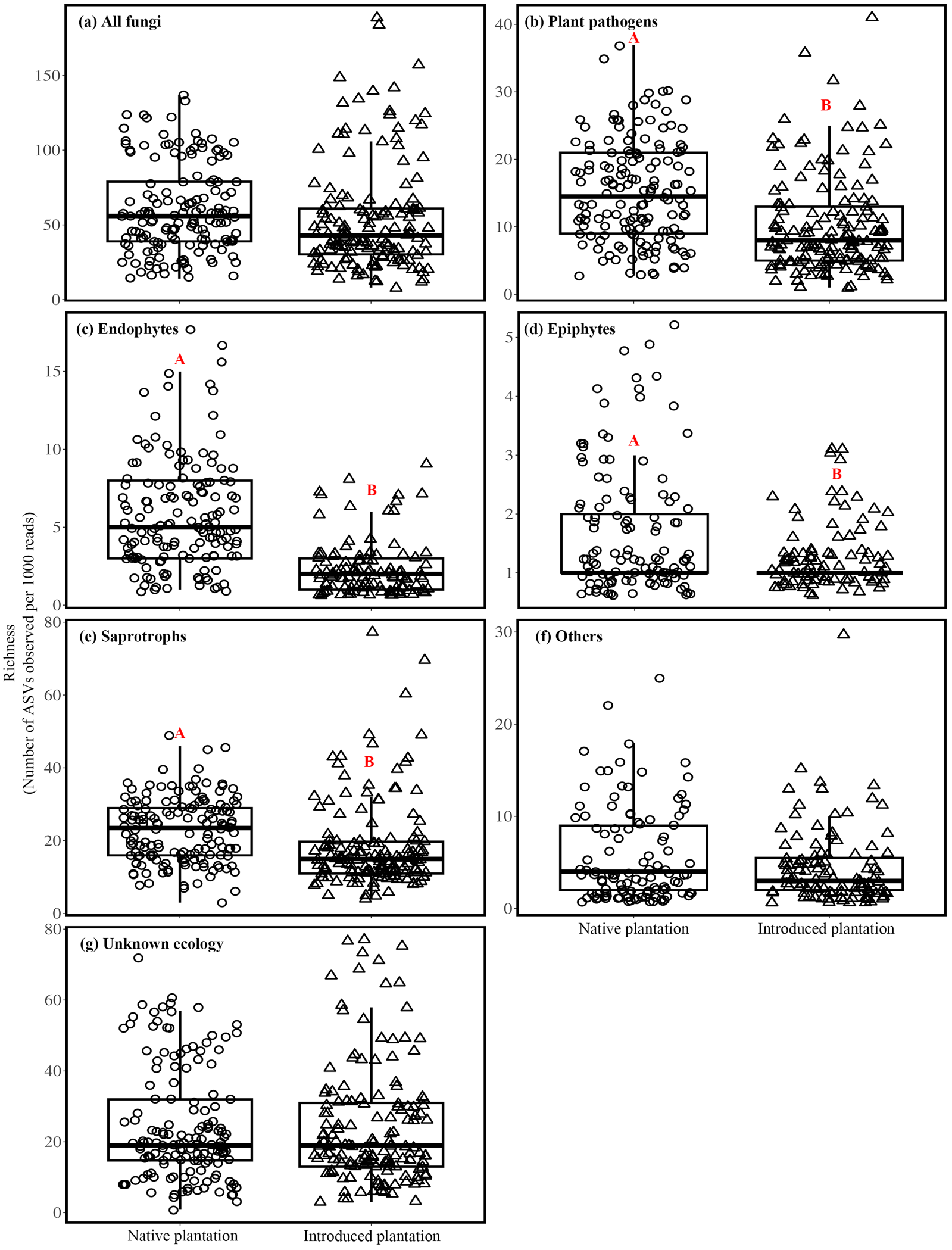
Figure S2.** Amplicon Sequence Variants (ASVs) richness of fungal communities associated with *Pinus contorta* needles in native (i.e. Canada and the USA) and introduced (i.e., Sweden and Patagonia) plantations. Richness is presented as the average rarified richness for each sample. Results from the corresponding Welch's t or Wilcoxon rank sum tests are reported in Table 2. Different letters above bars or across bar segments with the same shade indicate significant differences at α= 0.05, and the absence of letters indicates no significant difference.
