# Supplementary material for: Distinct foliar fungal communities in Pinus contorta across native and introduced ranges: evidence for context dependency of pathogen release
Source: Sci Rep. 2025 Mar 1;15:7273. doi: 10.1038/s41598-025-91639-z (PMC11873135; doi:10.1038/s41598-025-91639-z)
Supplement: Supplementary file 2 — Supplementary Material 2 [file 41598_2025_91639_MOESM2_ESM.docx]

**Supplementary Methods**

***DNA extraction, PCR, and PacBio***

In total DNA was extracted from 397 needle samples. The cleaned needles were homogenized using a sterile pestle or bead mill to break down cell walls and release genomic material. DNA was extracted from PL1 buffer (based on the CTAB procedure, 3% cetyltrimethylammonium bromide, 2 mM EDTA, 150 mM Tris-HCl and 2.5 M NaCl, pH 8) following the manufacturer’s protocol of NucleoSpin® Plant II kit (Macherey-Nagel GmbH & Co. KG, Düren, Germany). The presence and quantity of genomic DNA were checked using a NanoDrop 2000 spectrophotometer (Thermo Fisher Scientific, Dreieich, Germany), and the extracts were then stored at −20°C until needed for downstream PCR. Negative controls were included in all DNA extractions to monitor contamination.

PacBio Single Molecule, Real-Time (SMRT) sequencing of the tagged amplicons was performed at the SLU Metabarcoding Laboratory in Uppsala, Sweden. Blank and negative controls were included in all PCR analyses to monitor contamination. For each sample, PCR amplification of the ribosomal internal transcribed spacer (ITS) DNA was carried out in triplicate 50 μL reactions using the fungal-specific forward primer ITS1 (ACCWGCGGARGGATCATTA) and reverse primer ITS4 (TCCTCCGCTTATTGATATGC). The cycling conditions included an initial denaturation at 95°C for 5 minutes, followed by 21-35 cycles of denaturation at 95°C for 30 seconds, annealing at 55°C for 30 seconds, and elongation at 72°C for 1 minute. A final elongation step was performed at 72°C for 10 minutes.

PCR products were purified using AMPure XP beads (Beckman Coulter, USA) at a sample-to-bead-solution ratio of 1:1.8, and DNA was eluted in 65 μL of 5 mM Tris buffer (pH 8.5). Purification of the PCR products was performed using the Ezna Cycle-Pure Kit (Omega Biotek, USA), and their concentrations were measured according to the manufacturer’s protocol. The concentrations of the purified PCR products were determined using the Qubit HS kit (Thermo Fisher Scientific, USA), while absorbance ratios were evaluated using a Nanodrop spectrophotometer (Thermo Fisher Scientific, USA). The quality of the pools was assessed using the Agilent Bioanalyzer 2100 with a DNA 1000 chip (Agilent Technologies, USA). Subsequently, 100 ng of purified PCR product per sample was used for library preparation according to the PacBio protocol.
